# Intraparticle Electron Transfer for Long‐Lasting Tumor Chemodynamic Therapy

**DOI:** 10.1002/advs.202403935

**Published:** 2024-07-30

**Authors:** Jing Yu, Hongmeng Yan, Fan Zhao, Yao Ying, Wangchang Li, Juan Li, Jingwu Zheng, Liang Qiao, Wei Yang, Shenglei Che

**Affiliations:** ^1^ College of Materials Science and Engineering Research Center of Magnetic and Electronic Materials Zhejiang University of Technology Hangzhou 310014 China; ^2^ Department of Radiation Oncology The First Medical Center of Chinese PLA General Hospital Beijing 100853 China

**Keywords:** ^•^OH generation, chemodynamic therapy, GSH consumption, intraparticle electron transfer, long‐lasting cancer therapy

## Abstract

Chemodynamic therapy (CDT) is a novel tumor treatment method by using hydroxyl radicals (^•^OH) to kill cancer cells. However, its therapeutic effects are strictly confined by the short lifespan of ^•^OH and reduced ^•^OH generation speed. Herein, an effective CDT is achieved by both improving ^•^OH lifetime and long‐lasting generating ^•^OH through intraparticle electron transfer within heterogeneous nanoparticles (NPs). These heterogeneous NPs are composed of evenly distributed Cu and Fe_3_O_4_ (CFO NPs) with large interaction interfaces, and electrons tend to transfer from Cu to Fe_3_O_4_ for the appearance of ≡Cu^2+^ and increase in ≡Fe^2+^. The generated ≡Cu^2+^ can interact with GSH, which prolongs the lifespan of ^•^OH, produces ≡Cu^+^ for higher speed ^•^OH generation with H_2_O_2_, and induces cell ferroptosis for tumor therapy. The improved ≡Fe^2+^ can also improve the ^•^OH release under H_2_O_2_ until Cu is depleted. As a result, a sustainable ^•^OH generation is achieved to promote cell apoptosis for effective tumor therapy. Since H_2_O_2_ and GSH are only overexpressed at tumor, and CFO NPs can degrade in the tumor microenvironment, these NPs are with high biosafety and can be metabolized by urine. This work provides a novel biomaterial for effective cancer CDT through intraparticle electron transfer.

## Graphical abstract

Heterogeneous nanoparticles (NPs) composed of evenly distributed Cu and Fe_3_O_4_ (CFO NPs) are designed. Intraparticle electron transfer from Cu to Fe_3_O_4_ endows enrichment of ≡Fe^2+^ and appearance of ≡Cu^2+^ for long‐lasting ^•^OH generation and retention. These CFO NPs can induce apoptosis and ferroptosis for effective tumor therapy and can be metabolized by urine for good biocompatibility.

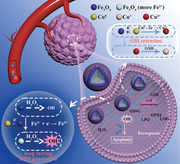

## 1 Introduction

Hydroxyl radicals (^•^OH) are a kind of highly active reactive oxygen species (ROS) that have dual effects on cells.^[^ ^1^ ^]^ By using ^•^OH as a therapeutic molecule, chemodynamic therapy (CDT) has emerged as a novel tumor therapy in the past decade,^[^ ^2^ ^]^ which benefits from the over‐expression of H_2_O_2_ at tumor and generation of ^•^OH through various Fenton‐like catalytic materials.^[^ ^3^ ^]^ However, the curative effects for CDT are still restricted by the low local ^•^OH concentration at the tumor, which can be ascribed to essentially short lifespan of ^•^OH (10^−9^ s), and scavenging of generated ^•^OH by highly expressed antioxidant substances, such as glutathione (GSH).^[^ ^4^ ^]^ This problem can be partly solved by depleting unwanted reductants by some oxidants, such as high valence variable metal ions and thiol oxidants, resulting in a long‐lived ^•^OH.^[^ ^5^ ^]^ However, this is a “passive” method that only works on the basis of efficient ^•^OH generation. If the ^•^OH yield is low, even if the total elimination of GSH will not help.

Actually, the primary barrier to effective CDT is the low ^•^OH yield at the tumor.^[^ ^6^ ^]^ This is because the present CDT agents are commonly Fe or Cu‐based materials, with ≡Fe^2+^ or ≡Cu^+^ optimal valence states.^[^ ^7^ ^]^ However, ≡Fe^2+^ and ≡Cu^+^ can quickly turn to inactive ≡Fe^3+^ and ≡Cu^2+^ during Fenton‐like reactions, leading to low‐speed ^•^OH generation.^[^ ^8^ ^]^ Therefore, it is challenging to continuously produce ^•^OH.^[^ ^9^ ^]^ Although some external fields can switch these ions back to the lower valence,^[^ ^10^ ^]^ the effects are temporary. The long‐lasting stay of CDT agents in the reduction state is still challenging.

**Scheme 1:**
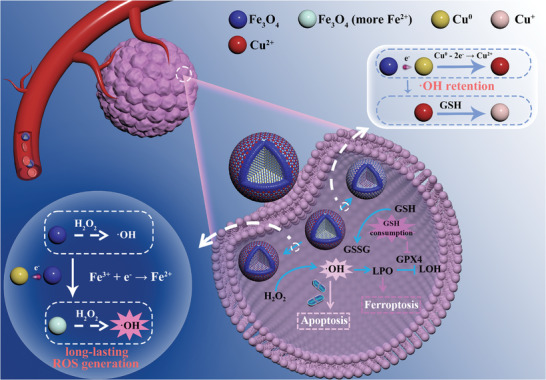
Internal redox reaction and antitumor mechanism of CFO NPs.

Herein, an effective CDT was achieved by using both long‐lasting ^•^OH generation strategy and GSH depletion based on intraparticle electron transfer within heterogeneous nanoparticles (NPs). As shown in **Scheme** **1**, the NPs were composed of Cu and Fe_3_O_4_, with these two components distributed evenly (CFO NPs) for higher interaction interfaces. Origin from the difference in work function between Cu and Fe_3_O_4_, electrons tend to transfer from Cu to Fe_3_O_4_ at the interfaces, resulting in the appearance of ≡Cu^2+^ in the Cu part and an increase in ≡Fe^2+^ in the Fe_3_O_4_ region. The ≡Cu^2+^ can interact with GSH, not only eliminating GSH for long‐lived ^•^OH, but also producing ≡Cu^+^ for higher speed ^•^OH generation in a Fenton‐like reaction. Meanwhile, the improved ≡Fe^2+^ portion endows NPs with better ^•^OH release under H_2_O_2_. The electron transfer products (≡Fe^2+^ and ≡Cu^2+^) can react with tumor‐abundant H_2_O_2_ and GSH, driving a long‐lasting electron transfer until the Cu was depleted. Thus, a sustainable ^•^OH generation was achieved in tumor cells, which promoted cell apoptosis through the activation of caspase‐3. Ferroptosis was also induced by the consumption of GSH and finally led to an effective tumor therapy. As both H_2_O_2_ and GSH are featured by tumor, and structures of CFO NPs collapsed during electron transfer for urine metabolism, these NPs are with high biosafety. Intraparticle electron transfer NPs designed in this work provide an effective and safe biomaterial for cancer CDT based on long‐lasting ^•^OH generation and GSH depletion.

## 2 Result and Discussion

### 2.1 Characterization of CFO NPs

**Figure 1:**
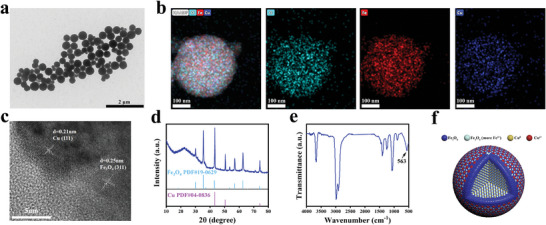
a) TEM image of CFO NPs, scale: 2 µm. b) Elemental mapping images of CFO NPs, scale: 100 nm. c) HADDF‐STEM images of CFO NPs, scale: 5 nm. d) XRD pattern of CFO NPs. e) FTIR spectrum of CFO NPs. f) Structure diagram of CFO NPs.

Heterogeneous NPs were synthesized by hydrothermal method. Transmission electron microscopy (TEM) was applied to observe the morphology of NPs. As shown in **Figure** **1**a, NPs exhibited a spherical structure with an average diameter of approximately 300 nm. Elemental mapping images showed the existence of Cu, Fe, and O, and all elements distributed evenly in NPs (Figure 1b). High‐resolution transmission electron microscope images (HRTEM) suggested d‐spacing of 0.21 and 0.25 nm appeared, which corresponded to (111) crystal face of Cu and (311) crystal face of Fe_3_O_4_ respectively (Figure 1c),^[^ ^11^ ^]^ indicating NPs were composed of Cu and Fe_3_O_4_. Results from the X‐ray diffraction (XRD) were in accordance with HRTEM, as all peaks fit well with Cu (PDF#04‐0836) and Fe_3_O_4_ (PDF#19‐0629) (Figure 1d). The concentrations of Cu and Fe elements in NPs were determined by an inductively coupled plasma optical emission spectrometer (ICP‐OES), which suggested the molar ratio of Fe_3_O_4_ to Cu in CFO NPs was about 1:1 (Table S1, Supporting Information). Fourier transform infrared spectroscopy (FTIR) is shown in Figure 1e. The appearance of the absorption peak at 563 cm^−1^, which could be ascribed to stretching vibration of the Fe─O,^[^ ^12^ ^]^ indicating Fe was in oxidation state. While no absorption peaks of Cu─O were observed, further suggesting that Cu appeared as ≡Cu^0^. All the above results suggested that the synthesized consisted of Cu and Fe_3_O_4_, and these two components distributed evenly (named CFO NPs, Figure 1f). The stability of CFO NPs in different solvents was then analyzed, which suggested the size of CFO NPs would not obviously change in water, phosphate buffer saline (PBS), or culture medium (DMEM) within 7 d (Figure S1, Supporting Information). These CFO NPs were negatively charged in PBS with zeta potential of about −10.8 mV (Figure S2, Supporting Information).

**Figure 2:**
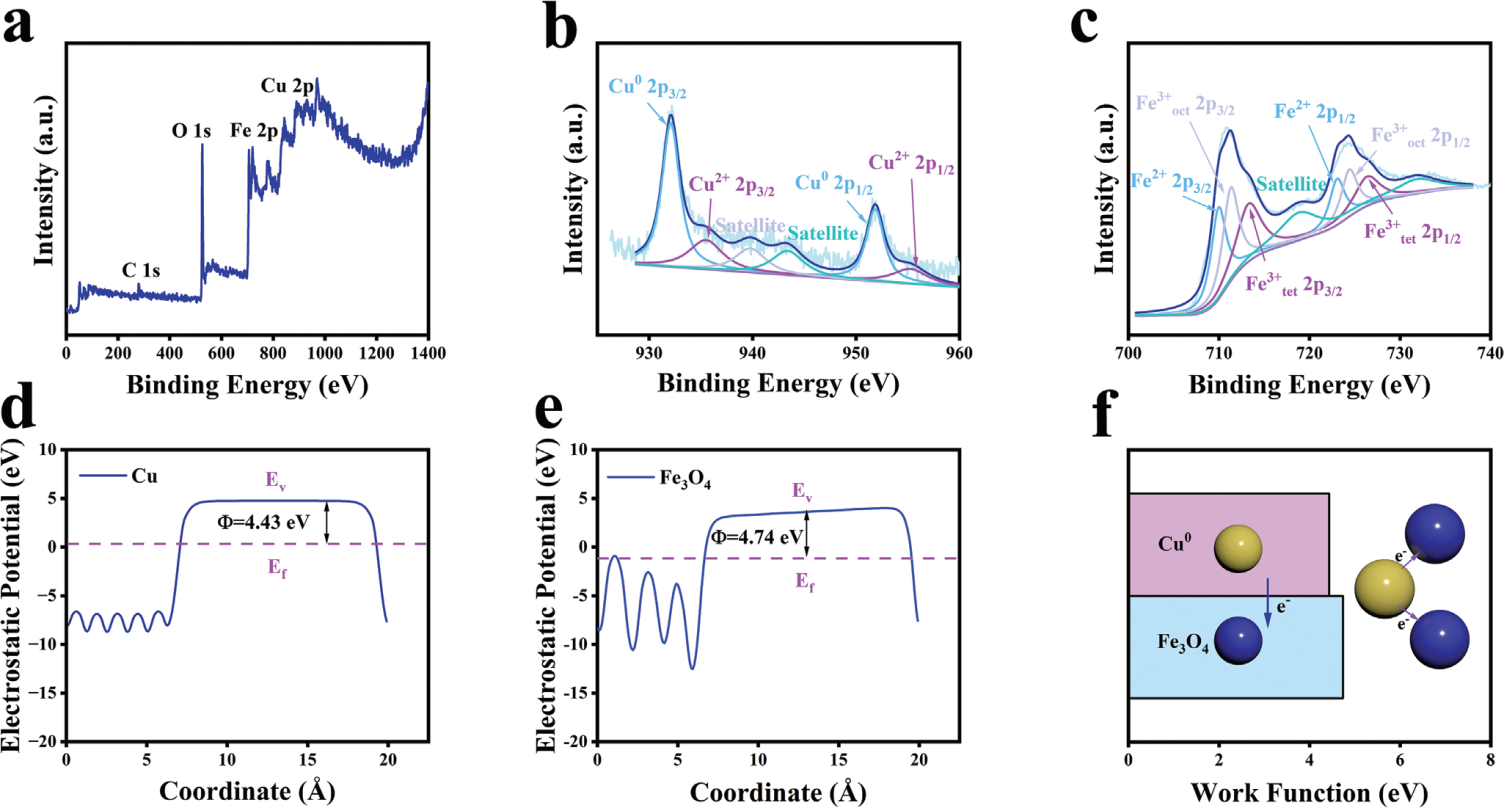
a) XPS spectrum of CFO NPs. High‐resolution XPS spectrum of CFO NPs: b) Cu 2p, c) Fe 2p. The average potential profiles of d) Cu^0^ and e) Fe_3_O_4_. f) Intraparticle electron transfer trend of CFO NPs.

X‐ray photoelectron spectroscopy (XPS) was further carried out to precisely investigate the valance of Cu and Fe (**Figure** **2**a–c). Peaks of Fe 2p at 709.6 and 711.2 eV were contributed to Fe^2+^ 2p_3/2_ and Fe^3+^ 2p_3/2_ respectively, showing Fe elements in CFO NPs were dominated by ≡Fe^2+^ and ≡Fe^3+^. Peaks of Cu 2p at 932.2 and 951.95 eV suggested Cu elements were primarily in the ≡Cu^0^ state. Interestingly, different from the results from FTIR, faint peaks at 933.8 and 953.55 eV were also observed at the Cu 2p spectrum, illustrating the presence of ≡Cu^2+^.^[^ ^13^ ^]^

This unexpected phenomenon could be ascribed to the intraparticle electron transfer from Cu to Fe_3_O_4_.^[^ ^9^ ^]^ According to the average potential profiles (Figure 2d,e and Figures S3 and S4, Supporting Information), the work function (*Φ*) of Cu is about 4.43 eV, which is lower than that of Fe_3_O_4_ (*Φ* = 4.74 eV). As a result, when Cu interacts with Fe_3_O_4_, electrons tend to transfer from Cu to Fe_3_O_4_ to equilibrate the Fermi level (EF) (Figure 2f), and ≡Cu^0^ with reduced electrons tends to appear as ≡Cu^2+^. After accepting electrons, the Fe_3_O_4_ domain in CFO NPs was enriched in ≡Fe^2+^, which can be confirmed by the improved signal in electron paramagnetic resonance (EPR) spectra of ≡Fe^2+^ (Figure S5, Supporting Information).

Since elemental mapping images suggested that Cu and Fe_3_O_4_ were distributed evenly in CFO NPs, the interaction interface between Cu and Fe_3_O_4_ is larger than other heterogeneous structures such as dimers and core–shell structure. Therefore, intraparticle electron transfer within CFO NPs is strong, which is beneficial for the design of applications of CFO NPs based on electron transfer.

### 2.2 ^•^OH Generation

**Figure 3:**
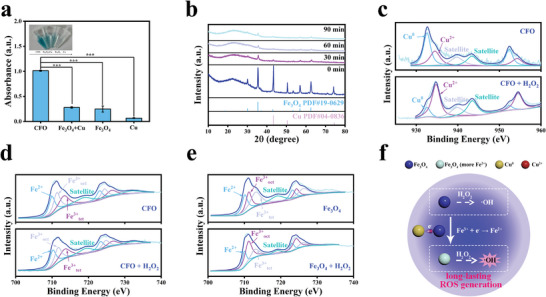
a) ^•^OH generation of different materials at pH 4.5, *n* = 3, ***p < 0.001. b) XRD pattern of CFO NPs after incubating with H_2_O_2_. High‐resolution XPS spectrum of different NPs after incubating with H_2_O_2_ for 30 min: c) CFO NPs, Cu 2p. d) CFO NPs, Fe 2p. e) Fe_3_O_4_ NPs, Fe 2p. f) Schematic diagram of electron transfer for long‐lasting ^•^OH generation.

This intraparticle electron transfer within CFO NPs can increase the ratio of ≡Fe^2+^ in Fe_3_O_4_ by obtaining electrons. As a result, ^•^OH generation by CFO NPs with H_2_O_2_ would be improved, as ≡Fe^2+^ is dominant for a Fenton‐like reaction. EPR spectra showed a clear symmetrical distribution of the peak, and the peak area ratio of 1:2:2:1, indicating that ROS generated by CFO NPs was ^•^OH (Figure S6, Supporting Information). By using 3,3′,5,5′‐tetramethylbenzidine (TMB) as an indicator, CFO NPs showed an obvious absorption peak at 652 nm (**Figure** **3**a and Figure S7, Supporting Information).^[^ ^14^ ^]^ In contrast, Fe_3_O_4_ NPs alone had with much lower absorbance, and the absorbance for Cu was indiscernible. Interestingly, the absorption peak for CFO NPs was even much higher than the mixture for Fe_3_O_4_ and Cu, indicating that intraparticle electron transfer plays an important role in improving Fenton‐like reaction. The acceleration of ^•^OH generation was further confirmed by investigating the ^•^OH generation rate, which showed that compared with Fe_3_O_4_ NPs, CFO NPs have a significantly improved ^•^OH generation speed, evidencing the long‐lasting ^•^OH generation (Figure S8, Supporting Information).

The acceleration of the Fenton‐like reaction can be ascribed to the transformation of ≡Fe^2+^ to ≡Fe^3+^ during ^•^OH generation, breaking the Fermi charge equilibrium within CFO NPs, and continuously providing electrons by Cu to Fe_3_O_4_. To verify this consumption, XRD of CFO NPs after different Fenton‐like reaction times was carried out. As shown in Figure 3b, peaks of Cu gradually disappeared, evidencing the sustaining intraparticle reactions within CFO NPs. XPS was also used to investigate the variation in valence during a Fenton‐like reaction. As shown in Figure 3c,d and **Table** **1**, after incubating with H_2_O_2_ for 30 min, peaks for ≡Cu^0^ decreased from 60.9% to 18.3% and the signal of ≡Cu^2+^ increased from 39.1% to 81.7% in CFO NPs, proving Cu was an electron donor for ^•^OH generation. Peaks for ≡Fe^2+^ in CFO NPs remained almost unchanged, while exhibiting a downward trend in bare Fe_3_O_4_ NPs (Figure 3e and Table 1). This is because ≡Fe^2+^ consumed during ^•^OH generation in CFO NPs can be immediately replenished by accepting electrons from ≡Cu^0^, leading to the ≡Fe^2+^ in CFO NPs remaining in a high concentration for sustainable Fenton‐like reaction (Figure 3f and Figure S9, Supporting Information). The above results suggested that CFO NPs have great potential in long‐term ^•^OH generation in H_2_O_2_‐rich tumor microenvironment for effective cancer therapy.

**Table 1:** Percentage of various ions in CFO NPs and Fe_3_O_4_ NPs after incubating with H_2_O_2_ for 30 min.

	≡Fe^2+^ [%]	≡Fe^3+^ [%]	≡Cu^0^ [%]	≡Cu^2+^ [%]
CFO	32.4	67.6	60.9	39.1
CFO+H_2_O_2_	33.7	66.3	18.3	81.7
Fe_3_O_4_	29.5	70.5	/	/
Fe_3_O_4_+H_2_O_2_	24.1	75.9	/	/

### 2.3 GSH Consumption

**Figure 4:**
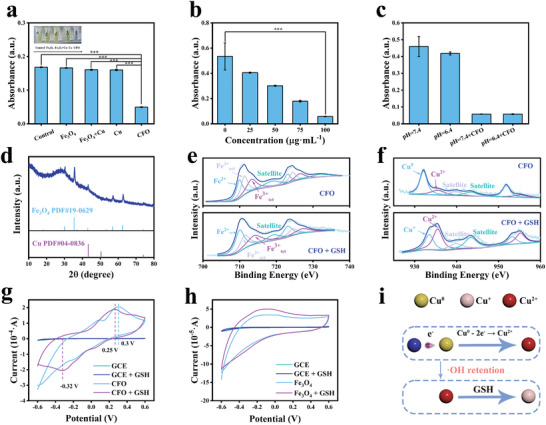
a) Absorptions of different NPs with DTNB at wavelength of 412 nm. Inset is the photograph of the result solution. Origin GSH concentration was 1 × 10^−3^ m. *n* = 3, ***p < 0.001. b) GSH consumption of CFO NPs in different concentrations, *n* = 3, ***p < 0.001. c) GSH consumption of CFO NPs in different pH, *n* = 3. d) XRD pattern of CFO NPs after GSH incubating. e,f) High‐resolution XPS spectrum of CFO NPs after GSH incubating for 24 h: e) Fe 2p, f) Cu 2p. CV curves of g) CFO NPs and h) Fe_3_O_4_ NPs. i) Schematic illustration of GSH consumption for ^•^OH retention.

The intraparticle electron transfer can also be used to consume GSH. By using 5,5′‐dithiobis‐(2‐nitrobenzoic acid) (DTNB) as a GSH indicator,^[^ ^15^ ^]^ CFO NPs were found to be able to dramatically remove GSH, while the addition of Fe_3_O_4_ NPs or Cu NPs could hardly decrease GSH content (**Figure** **4**a and Figure S10, Supporting Information), suggesting the elimination of GSH by CFO NPs was not the result of the direct reaction between these components. In addition, the GSH elimination efficiency only relied on the concentration of NPs rather than pH value (Figure 4b,c and Figure S11, Supporting Information), indicating this effect was originating from CFO NPs.

To investigate the mechanism of GSH elimination by CFO NPs, the XRD pattern of CFO NPs incubating with GSH for 24 h was carried out. As shown in Figure 4d, peaks of Cu almost disappeared, while the signals from Fe_3_O_4_ remained unchanged. It is astonishing that GSH can interact with Cu rather than Fe_3_O_4_. Previous results from Figure 4a have already confirmed Cu itself cannot react with GSH. To explain this phenomenon, the UV–Vis absorption spectrum of the mixture of Fe_3_O_4_ and Cu with DTNB was tested (Figure 4a and Figure S10, Supporting Information). Interestingly, even the mixture of Fe_3_O_4_ and Cu NPs is difficult in removing GSH, implying the GSH consumption of CFO NPs might originate from the interfacial interaction of NPs. XPS of CFO NPs after reacting with GSH was then analyzed. As suggested in Figure 4e,f and **Table** **2**, peaks for ≡Cu° completely disappeared, the intensity of peak for ≡Cu^2+^ and ≡Fe^2+^ was improved, and a decrease in ≡Fe^3+^ was observed. In addition, a new peak at 932.7 eV which was attributed to Cu^+^ 2p3/2 appeared, indicating the formation of ≡Cu^+^. The generation of ≡Cu^+^ after the addition of GSH was further confirmed by the EPR spectra of Cu^+^ (Figure S12, Supporting Information). Therefore, we assumed that GSH was consumed during the reduction of ≡Cu^2+^ to ≡Cu^+^, and the presence of ≡Cu^0^ promoted this process by improving ≡Cu^2+^ concentration through electron transfer.^[^ ^13^ ^]^

**Table 2:** Percentage of various ions in CFO NPs and Fe_3_O_4_ NPs after incubating with GSH.

	≡Fe^2+^ [%]	≡Fe^3+^ [%]	≡Cu^0^ [%]	≡Cu^+^ [%]	≡Cu^2+^ [%]
CFO	32.4	67.6	72.8	/	27.2
CFO+GSH	44.1	55.9	/	39.8	60.2

To prove this assumption, cyclic voltammetry (CV) was used to explore the redox reaction within CFO NPs.^[^ ^16^ ^]^ As shown in Figure 4g,h and Figures S13 and S14 (Supporting Information), in comparison with Fe_3_O_4_ NPs, whose curves were smooth, CFO NPs presented an oxidation peak at about 0.3 V, which is due to the oxidation of ≡Cu^0^ to ≡Cu^2+^ during the electron transfer process. The oxidation peak of CFO NPs shifted to 0.25 V after adding GSH, while no obvious distinctions were observed before and after the addition of GSH by Fe_3_O_4_ NPs, indicating that GSH can reduce the over‐potential to accelerate the intraparticle electron transfer within CFO NPs. This phenomenon can explain the results from XPS, where peaks for ≡Cu^2+^ and ≡Fe^2+^ were increased and ≡Fe^3+^ decreased after the addition of GSH. In addition, an obvious reduction peak of CFO NPs at −0.32 V was observed upon the apply of GSH, proving the reduction of ≡Cu^2+^ to ≡Cu^+^.

Taking all the results above, we can conclude that the GSH consumption by CFO NPs was mainly due to the reaction with ≡Cu^2+^, resulting in the decrease in ≡Cu^2+^ concentration initially. As ≡Cu^2+^ was generated during the electron transfer from Cu to Fe_3_O_4_, the cut‐down of the reaction product can speed up the electron transfer process, leading to the exhaustion of ≡Cu^0^ and increasing of ≡Cu^2+^ for further GSH depletion (Figure 4i and Figure S15, Supporting Information). In addition, apart from the GSH decreasing, the production of ≡Cu^+^ under GSH can further improve the Fenton‐like activity of CFO NPs for more efficient ^•^OH generation (Figure S16, Supporting Information).

### 2.4 In Vitro Therapy

Since both H_2_O_2_ and GSH are characterized by overexpression in tumor cells, these CFO NPs should possess a synergetic tumor therapy with high efficiency.

**Figure 5:**
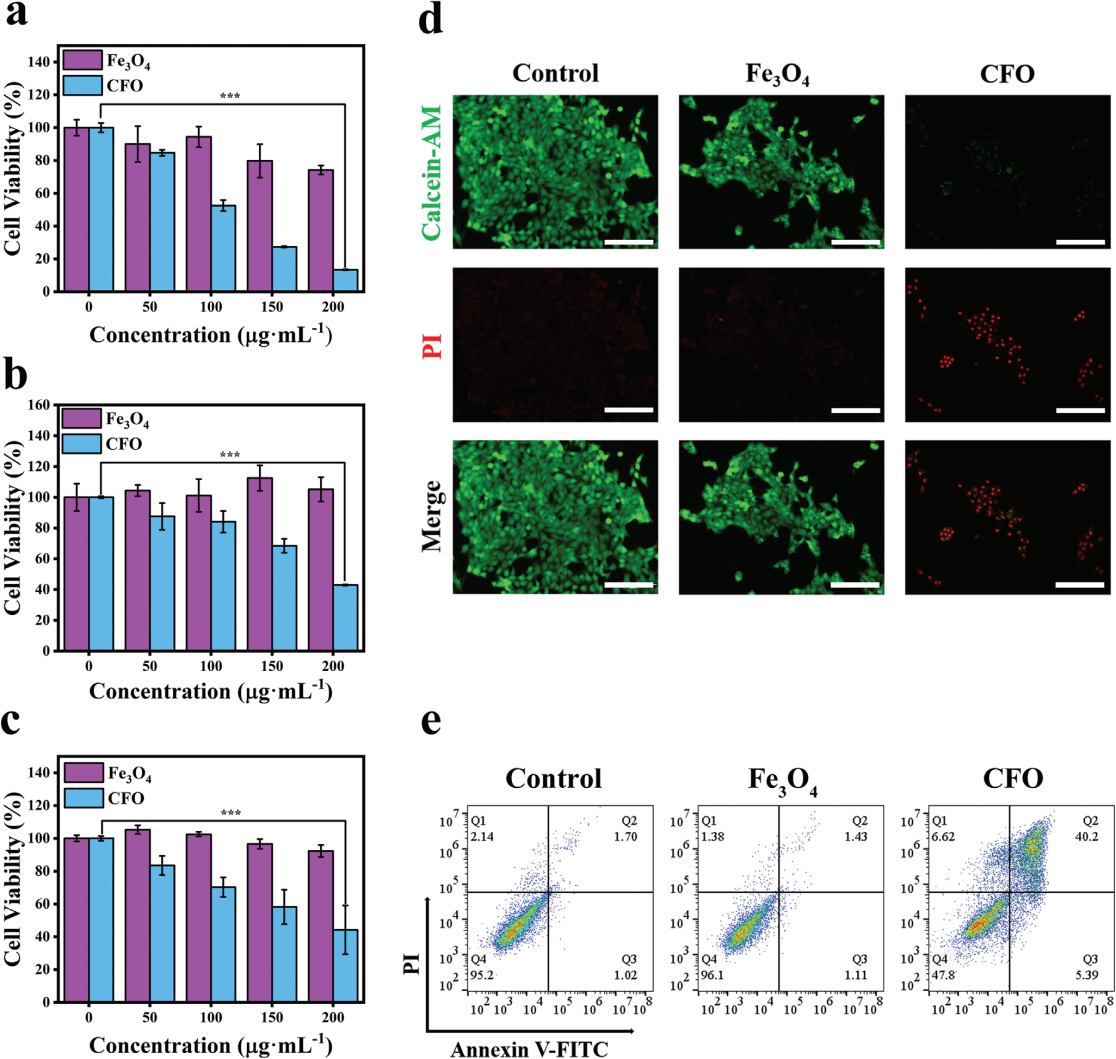
Viability of a) 4T1, b) CT26, and c) HepG2 cells incubated with different concentrations of CFO NPs, *n* = 6, ***p < 0.001. d) Fluorescence images of 4T1 cells stained with calcein‐AM/PI after incubating with different NPs, in different magnification, scale: 150 µm. e) Flow cytometry analysis of 4T1 cells after incubating with different NPs.

Methyl thiazolyl tetrazolium (MTT) assay was adopted to characterize the cytotoxicity of CFO NPs towards cancer cells. As shown in **Figure** **5**a–c, CFO NPs revealed an evident toxicity to 4T1, CT26, and HepG2 cells after incubation for 24 h, with cell viability decreased by 13.4%, 42.9%, and 44.1%, respectively, when the concentration reached to 200 µg mL^−1^, while Fe_3_O_4_ NPs alone showed much lower cytotoxicity. Results from living/dead cells staining assay were in accordance with this phenomenon. A much higher red color from PI was observed from CFO NPs compared with Fe_3_O_4_ NPs, and infinitesimal green color was emitted by staining with calcein‐AM (Figure 5d). Flow cytometry data showed a significant proportion of apoptotic cells was yielded with the addition of CFO NPs (Figure 5e), confirming the superior antitumor efficacy of CFO NPs.

### 2.5 Mechanism of CFO NP‐Induced Cell Death

**Figure 6:**
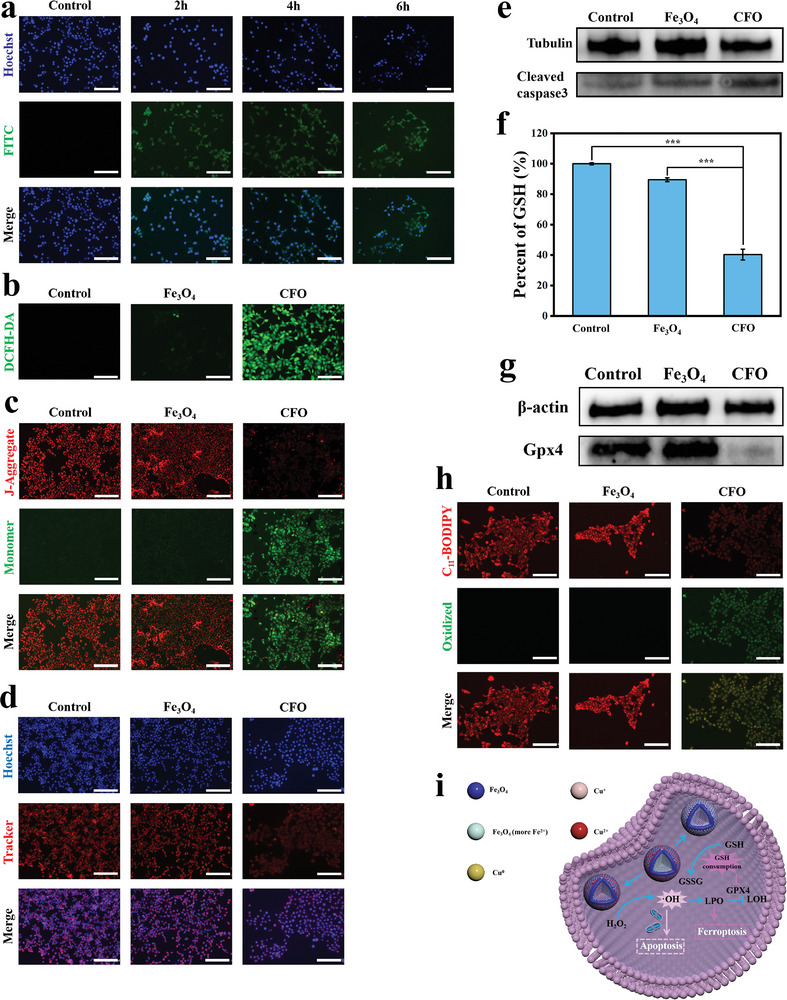
a) Fluorescence images of 4T1 cells incubated with FTIC labeled CFO NPs for different time, scale: 150 µm. b) Fluorescence images of DCFH‐DA stained 4T1 cells after incubating with different NPs, scale: 150 µm. c) Fluorescence images of JC‐1 stained 4T1 cells after incubating with different NPs, scale: 150 µm. d) Fluorescence images of Mito‐Tracker Red stained 4T1 cells after incubating with different NPs, scale: 150 µm. e) Western blot analysis on the expressions of tubulin and cleaved caspase3 after incubating with different NPs. f) Concentration of GSH in 4T1 cells after incubating with Fe_3_O_4_ NPs and CFO NPs for 12 h, ***p < 0.001. g) Western blot analysis on the expressions of β‐actin and GPX4 after incubating with different NPs. h) Fluorescence images of 4T1 cells stained with C_11_‐BODIPY after incubating with different NPs, scale: 150 µm. i) Schematic illustration of the CFO NPs‐induced cell death mechanism.

Results from flow cytometry indicated cell death pathway induced by CFO NPs was mainly apoptosis, which should interact within cells, cellular uptake was first monitored by using FITC‐labeled CFO NPs. As shown in **Figure** **6**a, the blue fluorescence of Hoechst was well surrounded by green fluorescence from FITC, and the intensity of green fluorescence was increased along with incubation time, indicating the cellular uptake of CFO NPs by tumor cells. Results from flow cytometry data also confirmed the cellular uptake of CFO NPs (Figure S17, Supporting Information).

Since ^•^OH generation is closely related to cell apoptosis, 2′,7′‐dichlorodihydrofluorescein diacetate (DCFH‐DA) was then applied to investigate the cellular ^•^OH. As demonstrated in Figure 6b, bright green fluorescence was emitted from CFO NPs treated cells, while almost no fluorescence was observed by Fe_3_O_4_ NPs treated group, indicating an improved ^•^OH concentration by CFO NPs treatment. High ^•^OH concentration can lead to severe oxidative stress for the emergence of mitochondrial dysfunction, which was assessed by using fluorescent probe JC‐1. As depicted in Figure 6c, CFO NPs triggered an enhancement of green fluorescence (abnormal mitochondria) and dim red fluorescence (healthy mitochondria), suggesting higher mitochondrial dysfunction was evoked. Mito‐Tracker Red CMXRos was further used to evident the change of mitochondrial membrane potential. Much weaker red fluorescence was observed with the addition of CFO NPs (Figure 6d), indicating the evocation of mitochondrial dysfunction. Since the caspase3 protein is a key link in the process of apoptosis, western blot (WB) was subsequently carried out, which suggested the expression of caspase3 was upregulation by CFO NPs, evidencing the promotion of apoptosis (Figure 6e and Figure S18, Supporting Information).^[^ ^17^ ^]^ Additionally, as H_2_O_2_ is specifically high in cancer cells, ^•^OH can only abundantly be produced within cancer cells by decomposing H_2_O_2_ with CFO NPs. As a result, when incubating CFO NPs with normal L929 cells, a much‐reduced cytotoxicity was observed (Figure S19, Supporting Information), demonstrating the potential biosafety of CFO NPs.

The evaluated intracellular ^•^OH stimulated by CFO NPs can be ascribed to the combination of rapid ^•^OH generation by CFO NPs with H_2_O_2_ and GSH depletion for higher ^•^OH retention. Thus, intracellular concentration of GSH was detected by DTNB. As expected, intracellular GSH treated with CFO NPs decreased by 60%, while only 11% GSH was consumed by incubation with Fe_3_O_4_ NPs (Figure 6f). The decrease in GSH concentration by CFO NPs can also break the cell protection from glutathione peroxidase 4 (GPX4).^[^ ^18^ ^]^ Therefore, the expression of GPX4 was investigated by WB, which revealed a remarkable downregulation of GPX4 by CFO NPs (Figure 6g). The reduced GPX4 can induce lipid peroxides (LPO), and hurt membrane architecture to induce cell ferroptosis. Then, C_11_‐BODIPY, an LPO indicator was selected to detect the level of LPO after incubation with CFO NPs, which showed a fluorescence change (Figure 6h). The green fluorescence turned brighter while the red fluorescence vanished, suggesting high concentration of LPO generated to induce ferroptosis. Lipidomic changes of 4T1 cells after NPs treatment also confirmed the improved LPO generation by CFO NPs (Figure S20, Supporting Information).

Rescue experiments that cell viabilities of CFO NPs incubated with free radical scavengers (NAC) and ferroptosis inhibitor (Fer‐1) were also studied, which showed an effective restoration of cell viability by using both NAC and Fer‐1 (Figure S21, Supporting Information), further indicating that both ^•^OH generation and GSH diminishing were the keys for the cytotoxicity of CFO NPs.

In conclusion, CFO NPs were cytotoxic toward cancer cells due to the effective ^•^OH generation and GSH inhibition. The consumption of tumor‐rich GSH on the one hand, could induce ferroptosis for cancer cell killing, on the other hand, was able to transform ≡Cu^2+^ to ≡Cu^+^ on CFO NPs for a better Fenton‐like reaction. Together with tumor‐abundant H_2_O_2_, which speeds up the electron transfer on CFO NPs, these NPs could effectively generate ^•^OH with the help of H_2_O_2_. In addition, the GSH diminishing makes ^•^OH stably presented. These CFO NPs are “zero‐waste” materials for effective and long‐lasting cancer therapy through both apoptosis and ferroptosis (Figure 6i).

### 2.6 Biosafety Evaluation

**Figure 7:**
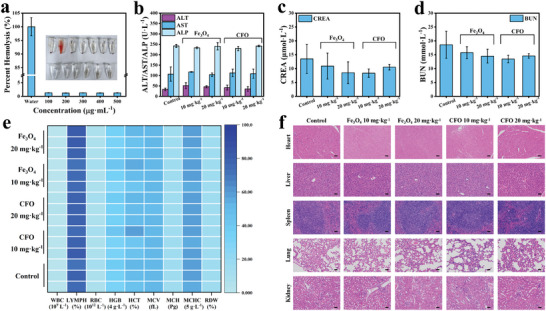
a) Hemolysis experiment with different concentrations of CFO NPs, *n* = 3. b) Liver function indices of mice, *n* = 3. Renal function indices of mice, c) CREA and d) BUN, *n* = 3. e) The hematological parameters of mice. f) Images of H&E staining of different organs, scale: 50 µm.

Inspired by the promising in vitro results, in vivo assays were then carried out. Since both H_2_O_2_ and GSH are tumor‐selective overexpressed, CFO NPs with H_2_O_2_ and GSH active should be biosafe. Hemocompatibility of CFO NPs was first assessed, which showed a low hemolysis rate (below 5%) within the range of 100–500 µg mL^−1^, suggesting that CFO NPs were with good blood compatibility (**Figure** **7**a). Blood biochemical and blood routine examinations were then analyzed. Indexes from liver function and kidney function were all within the normal range after injecting CFO NPs (Figure 7b–d), and blood routine examination results indicated CFO NPs would not induce abnormal physiological indexes (Figure 7e). The hematoxylin and eosin (H&E) staining were used to investigate injury of major organs after injection of CFO NPs (10 and 20 mg kg^−1^), and no pathological abnormalities were observed (Figure 7f), which further proved CFO NPs had good biocompatibility.

### 2.7 Biodistribution

**Figure 8:**
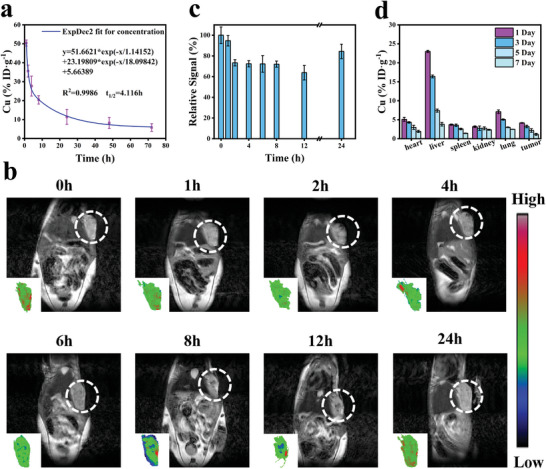
a) Pharmacokinetic parameters of CFO NPs, *n* = 3. b) Images of T_2_ MRI mapping after injecting CFO NPs in different times. c) Signal quantization of tumor location, *n* = 3. d) Biodistribution of CFO NPs at different times, *n* = 6.

Effectively delivering NPs to tumors is essential for cancer therapy. The pharmacokinetic parameters of CFO NPs were investigated by measuring Cu contents in plasma after intravenous (i.v.) injection. As shown in **Figure** **8**a, the blood circulation of CFO NPs was consistent with the two‐compartment pharmacokinetic model,^[^ ^19^ ^]^ which featured a blood circulation half‐time of 4.116 h. The relatively high blood circulation stability of CFO NPs might be ascribed to their large particle size and negative charge.^[^ ^20^ ^]^ As CFO NPs are superparamagnetism with magnetization saturation (*M*_s_) of 60.9 emu g^−1^ (Figure S22, Supporting Information), T_2_‐MRI was able to trace the biodistribution of NPs. CFO NPs were i.v. injected at a dose of 10 mg kg^−1^, and MR images were taken before and 1 d postinjection. As shown in Figure 8b,c, a strong hypo‐intensity in T_2_‐MRI was induced after injection of CFO NPs, with the signal intensity significantly decreasing to 63.8% 12 h postinjection. The signal recovered to 84.4% 24 h later, indicating that CFO NPs can partly be metabolized. We ascribed this interesting phenomenon to the degradation of NPs. When CFO NPs were incubated in the tumor‐microenvironment with pH of 6.4 and GSH of 1 × 10^−3^ m for 24 h, NPs with spherical structure collapsed into small NPs (Figures S23 and S24, Supporting Information), endowing the potential for renal metabolism. Afterward, ICP‐OES was used to test the NPs concentration in different organs within one week by testing the Cu content. As shown in Figure 8d, NPs gradually decreased at the tumor, while their contents in the kidney remained almost unchanged, evidencing the possible metabolism by renal. Therefore, the metabolism of CFO NPs in vivo was determined by detecting the Cu content in the urine by ICP‐OES. The results showed that the Cu content in the urine increased within the first 1 d and then decreased, further verifying the urine metabolism of CFO NPs (Figure S25, Supporting Information).

### 2.8 In Vivo Therapy

**Figure 9:**
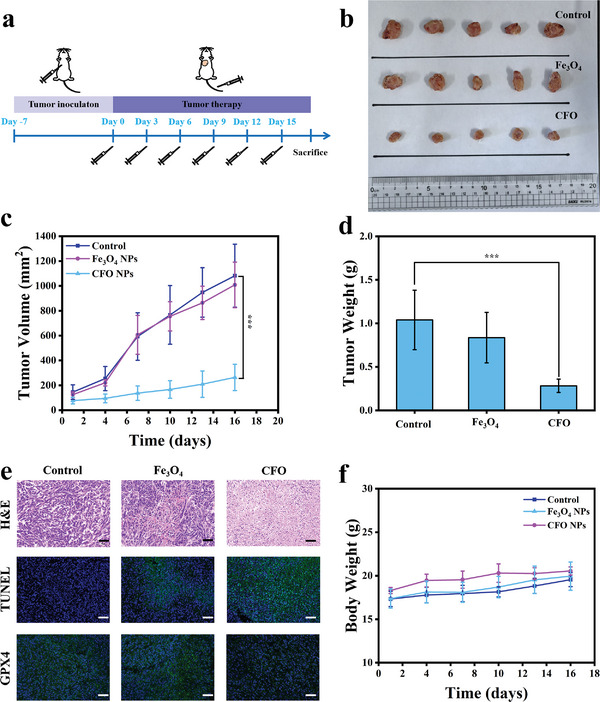
a) Experimental scheme of therapy in vivo. b) The photo of 4T1 tumor after experimental period. c) The tumor volume of different groups, *n* = 5, ***p < 0.001. d) The tumor weight of different groups, *n* = 5, ***p < 0.001. e) Images of H&E staining, TUNEL staining, and GPX4 staining of tumor, scale: 50 µm. f) The change of body weight of different groups, *n* = 5.

Antitumor efficacy of CFO NPs was finally assessed. The mice were randomly divided into three groups: control, i.v. injection of Fe_3_O_4_ NPs (10 mg kg^−1^) and i.v. injection of CFO NPs (10 mg kg^−1^), and NPs were injected every 3 d. The tumor diameters and body weights were measured on the next day post‐treatment (**Figure** **9**a). As shown in Figure 9b–d and Figures S26–S28 (Supporting Information), the tumor growth was apparently suppressed by CFO NPs, while no significant delay in tumor growth was observed in mice treated with Fe_3_O_4_ NPs or control. As shown in Figure 9e, hematoxylin and eosin (H&E) staining results suggested that severe karyolysis and cytosolic degradation were observed in CFO NPs treated tumor, confirming the tumor cell destruction by CFO NPs. TUNEL fluorescence staining showed obvious green fluorescence was emitted from CFO NPs treated tumor, suggesting extensive apoptosis of tumor was induced. GPX4 fluorescence staining with weak green light indicated that the expression of GPX4 was significantly down‐regulated by CFO NPs. These results further verified that CFO NPs could inhibit tumor growth by triggering both apoptosis and ferroptosis of tumor cells in vivo. The remarkable tumor inhibition of CFO NPs was origin from the effective ^•^OH generation. The ^•^OH content at the tumor was measured by the dihydroethidium (DHE) staining 7 and 14 d postinjection of CFO NPs, which showed an improvement in red fluorescence and a decrease of blue fluorescence (Figure S29, Supporting Information). All mice remained at normal body weight during the whole treatment period (Figure 9f), further indicating CFO NPs had high biosafety.

## 3 Conclusion

In summary, CFO NPs composed of evenly distributed Cu and Fe_3_O_4_ were synthesized by solvothermal method. Benefiting from the numerous interfaces and intraparticle electron transfer from Cu to Fe_3_O_4_ at the interfaces, ≡Cu^2+^ appeared at the Cu region and ≡Fe^2+^ increased in the Fe_3_O_4_ part. The generated ≡Cu^2+^ interacted with GSH, not only prolonging the lifespan of ^•^OH, but also producing ≡Cu^+^. Together with an improved ≡Fe^2+^ portion, these CFO NPs are with highly efficient ^•^OH generation under H_2_O_2_ until Cu is depleted. The long‐lasting ^•^OH release in tumor cells promoted cell apoptosis by activating caspase‐3, and the consumption of GSH‐induced cell ferroptosis. Therefore, effective tumor therapy could be activated by i.v. injection of CFO NPs. These NPs degraded at tumor microenvironment, making them urine metabolism after treatment for high biosafety. This work provided a novel biomaterial for effective and safe cancer CDT based on intraparticle electron transfer.

## 4 Experimental Section

### Material

Iron trichloride (FeCl_3_, CP, ≥98%) was purchased from Sinopharm Chemical Reagent Corporation. Cupric chloride dihydrate (CuCl_2_·2H_2_O, AR), glutathione (reduced) (GSH, 98%), and 5,5′‐dithio bis‐(2‐nitrobenzoic acid) (DTNB, 98%) were purchased from Aladdin. Sodium acetate trihydrate (C_2_H_3_O_2_Na·3H_2_O, AR, 99%) was purchased from Macklin. Ethylene glycol (HOCH_2_CH_2_OH, AR, ≥99.5%), hydrogen peroxide (H_2_O_2_, GR, ≥30%), and sodium citrate tribasic dihydrate (HOC(COONa)(CH_2_COONa)_2_·2H_2_O, AR, ≥99.0%) were purchased from Greagent. 3′,6′‐Dihydroxy‐5‐isothiocyanato‐3H‐spiro[isobenzofuran‐1,9′‐xanthen]−3‐one (FITC) was purchased from Solarbio. Methylthiazolyldiphenyl‐tetrazolium bromide (MTT, 98%), calcein/PI assay kit, annexin V‐FITC apoptosis detection kit, reactive oxygen species assay kit, total glutathione assay kit, mitochondrial membrane potential assay kit with JC‐1, mito‐tracker red CMXRos, and Hoechst 33258 were purchased from Beyotime Biotechnology.

### Synthesis of the CFO NPs

15 mmol FeCl_3_ and 7.5 mmol CuCl_2_·2H_2_O were dissolved in 100 mL EG, and stirred at 60 °C for 3 h, followed by the addition of 100 mmol sodium acetate trihydrate and 8 mg sodium citrate tribasic dihydrate stirred at 60 °C for 40 min. Last, the mixture was transferred into Teflon reactor and heated to 200 °C for 12 h.

### Synthesis of the Fe_3_O_4_ NPs

15 mmol FeCl_3_ was dissolved in 100 mL EG, and stirred at 60 °C for 3 h, followed by the addition of 100 mmol sodium acetate trihydrate and 8 mg sodium citrate tribasic dihydrate stirred at 60 °C for 40 min. Last, the mixture was transferred into Teflon reactor and heated to 200 °C for 12 h.

### Characterization

Transmission electron microscopy (TEM, HITACHI HT7700 EXALENS) and X‐ray photoelectron spectroscopy (XPS, Thermo Scientific K‐Alpha) were employed to observe the morphology and investigate the chemical composition of CFO NPs, respectively. Additionally, X‐ray diffraction (XRD) patterns were recorded using a PANalytical Empyrean powder diffractometer with Cu Kα radiation (*λ* = 0.1541 nm). The diffractometer operated at a working voltage of 40 kV and a working current of 40 mA. The patterns were collected over a 2*θ* range from 10° to 80° with a step size of 0.0262°.

### ^•^OH Production

CFO NPs (1 mg mL^−1^, 100 µL), H_2_O_2_ (0.05, 0.1, 0.15, 0.2, 0.25 m) and TMB (10 mg mL^−1^, 20 µL) were dissolved into PBS (pH = 6.4, pH = 5.4) or HAC buffer (pH = 4.5), and stirred at 40 °C for 2 min (total volume 2 mL). The absorbance of 652 nm was measured by microplate reader spectrophotometer. When Cu, Fe_3_O_4_, Fe_3_O_4_ + Cu, and CFO were used for comparison, the material concentration was 1 mg mL^−1^, 100 µL, and the other conditions remained unchanged.

### DFT Calculations

The DFT simulations were conducted using the Vienna ab initio simulation package (VASP) code, employing the projector augmented wave (PAW) methodology. To accurately compute the local potential at the material's surface, a vacuum layer of 20 Å was incorporated to mitigate spurious interactions. Additionally, the plane‐wave basis cut‐off energy was established at 450 eV for precision purposes.

### GSH Catalysis Assay

CFO NPs (1 mg mL^−1^, 50, 100, 150, and 200 µL) and GSH (10 × 10^−3^ m, 200 µL) were dissolved into PBS (pH = 7.4/6.4), with total volume of 2 mL. After 24 h and followed by centrifugation, DTNB (20 µL) solution was added to the supernatant. The absorbance at 412 nm was measured by microplate reader spectrophotometer. When Cu, Fe_3_O_4_, Fe_3_O_4_ + Cu, and CFO were used for comparison, the material concentration was 1 mg mL^−1^, 100 µL, and the other conditions remained unchanged.

### Electrochemical Analysis

5 mg CFO NPs was mixed with 5 mg X‐72 carbon black in the solution containing 1 mL ethanol and 1 mL H_2_O and sonicated for 30 min, and then, 50 µL Nafion was added and sonicated for another 10 min. The working electrode was prepared by dripping the catalysis (5 µL) onto the GCE electrode. Ag/AgCl and platinum wire were chosen as reference and counter electrodes, respectively. The electrolyte solution was PBS (pH = 6.5) with or without GSH (1 × 10^−3^ m).

### Cell culture

Zhejiang Provincial People's Hospital provides mouse breast cancer cells (4T1 cells), mouse colon cancer cells (CT26 cells), and human hepatocellular carcinomas cells (HepG2 cells). 10% fetal bovine serum (FBS) and 1% double antibody were added to DMEM medium. Cells were incubated at 37 °C and 5% CO_2_ atmosphere.

### Uptake Assay

4T1 cells (10^5^ per well) were seeded into 12‐well plates for 24 h. And then, FITC‐labeled CFO NPs (200 µg mL^−1^) were added into a medium and incubated with cells for 2, 4, or 6 h. Next, Hoechst 33258 was used to stain the nucleus for 30 min. Fluorescence microscope was used to acquire fluorescence images.

### MTT Assay

4T1 cells (5000 per well) were seeded into 96‐well plates for 24 h. Different concentrations of CFO NPs (50, 100, 150, 200 µg mL^−1^) and Fe_3_O_4_ NPs (50, 100, 150, 200 µg mL^−1^) were added into DMEM medium (without 10% FBS) and incubated with cells for 24 h. Supernatant was discarded, and then, adding MTT (5 mg mL^−1^, MTT:DMEM = 1:5, 100 µL) in wells for 4 h. The absorbance of 490 nm was measured by ELISA reader (SpectraMax 190).

### Live/Dead Staining Assay

4T1 cells (2 × 10^5^ per well) were seeded into six‐well plates for 24 h. CFO NPs (200 µg mL^−1^) and Fe_3_O_4_ NPs (200 µg mL^−1^) were incubated with cells for 24 h. Calcein‐AM and PI were incubated with cells for 30 min. Fluorescence microscope was used to acquire fluorescence images.

### Apoptosis Assay

4T1 cells (2 × 10^5^ per well) were seeded into six‐well plates for 24 h. CFO NPs (200 µg mL^−1^) and Fe_3_O_4_ NPs (200 µg mL^−1^) were incubated with cells for 16 h. Trypsin was used to digest cells. Then, Annexin V‐FITC and PI were incubated with cells for 30 min. Result was followed by flow cytometry analysis (BECKMAN COULTER, CytoFLEX S).

### Intracellular ^•^OH Assay

4T1 cells (2 × 10^5^ per well) were seeded into six‐well plates for 24 h. CFO NPs (200 µg mL^−1^) and Fe_3_O_4_ NPs (200 µg mL^−1^) were incubated with cells for 6 h. Cells were stained by 2′,7′‐dichlorodihydrofluorescein diacetate (DCFH‐DA) for 30 min. Fluorescence microscope was used to acquire fluorescence images.

### Mitochondrial Membrane Potential Assay

4T1 cells (2 × 10^5^ per well) were seeded into six‐well plates for 24 h. CFO NPs (200 µg mL^−1^) and Fe_3_O_4_ NPs (200 µg mL^−1^) were incubated with cells for 12 h. Cells were stained by JC‐1 for 30 min, or stained by Mito‐Tracker Red CMXRos for 30 min. After that, cells were stained by Hoechst 33258 for another 30 min. Fluorescence microscope was used to acquire fluorescence images.

### C_11_‐BODIPY Staining Assay

4T1 cells (2 × 10^5^ per well) were seeded into six‐well plates for 24 h. CFO NPs (200 µg mL^−1^) and Fe_3_O_4_ NPs (200 µg mL^−1^) were incubated with cells for 12 h. C_11_‐BODIPY was incubated with cells for 30 min. Fluorescence microscope was used to acquire fluorescence images.

### Western Blot Assay

4T1 cells (2 × 10^5^ per well) were seeded into six‐well plates for 24 h. CFO NPs (200 µg mL^−1^) and Fe_3_O_4_ NPs (200 µg mL^−1^) were incubated with cells for 12 h. Using trypsin to digest cells and collect cells. Adding loading buffer, followed by ultrasonic treatment, and metal bath (95 °C), standard western blot analysis was finally used to examine key markers GPX4 for ferroptosis and cleaved caspase3 for apoptosis.

### Animal Modal

All animal experiments were approved by the Animal Ethics Committee of Zhejiang University of Technology (MGS20230730109). Female Balb/c mice of 4–6 weeks were purchased from Qizhen Laboratory Animal Technology Co., Ltd. 5 × 10^6^ 4T1 cells were suspended in 0.1 mL sterile PBS for tumor inoculation.

### Biosafety Assay

Different concentrations of Fe_3_O_4_ NPs (10 mg kg^−1^, 20 mg kg^−1^, 100 µL), CFO NPs (10 mg kg^−1^, 20 mg kg^−1^, 100 µL) and PBS were injected into mice by intravenous injection. After 15 d, blood and organs (heart, liver, spleen, lung, kidney) were collected to evaluate blood chemistry and routine analysis, hematoxylin and eosin (H&E) staining.

### Biodistribution

Tail vein injection was performed on tumor‐bearing mice at different intervals (1, 3, 5, 7 d), CFO (10 mg kg^−1^, 100 µL). After 7 d, the mice were euthanized and the organs and tumors were collected to test the Cu ion content using ICP‐OES. The material concentration used in the MRI experiment was the same.

### In Vivo Therapy

Tumor‐bearing mice were assigned to three groups (*n* = 5): control, Fe_3_O_4_ (10 mg kg^−1^, 100 µL), and CFO (10 mg kg^−1^, 100 µL). NPs were injected into mice by intravenous injection per 3 d. And tumor volume (short diameter^2^ × long diameter/2) was calculated per 3 d and body weight was recorded at the same time. After 15 d, mice were killed, and tumors were collected to analyze H&E, TUNEL, and GPX4 staining.

## Conflict of Interest

The authors declare no conflict of interest.

## Supporting information

Supporting Information

## Data Availability

Research data are not shared.
