# Supplementary material for: Intraparticle Electron Transfer for Long‐Lasting Tumor Chemodynamic Therapy
Source: Adv Sci (Weinh). 2024 Jul 30;11(36):2403935. doi: 10.1002/advs.202403935 (PMC11423095; doi:10.1002/advs.202403935)
Supplement: Supplementary file 1 — Supporting Information [file ADVS-11-2403935-s001.docx]

Supporting Information

Intraparticle Electron Transfer for Long-Lasting Tumor Chemodynamic Therapy

Jing Yu*, Hongmeng Yan, Fan Zhao, Yao Ying, Wangchang Li, Juan Li, Jingwu Zheng, Liang Qiao, Wei Yang*, Shenglei Che*

**Table S1.** The concentration of Cu and Fe elements in CFO NPs.

|  | Fe  (mg·L^-1^) | Cu  (mg·L^-1^) |
| --- | --- | --- |
| CFO | 6.29 | 2.46 |


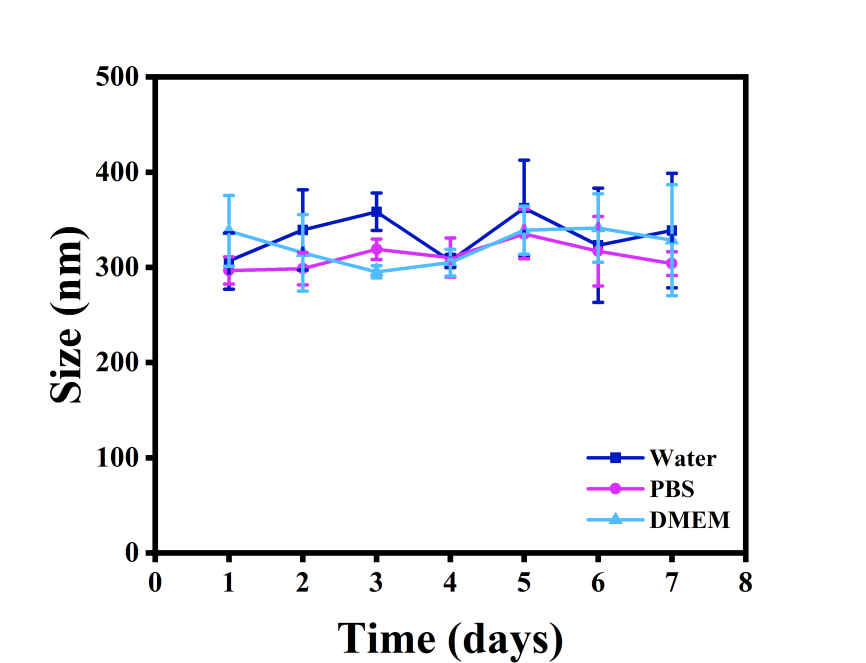


**Figure S1.** The change of the sizes of CFO NPs in different solution within 7 days.


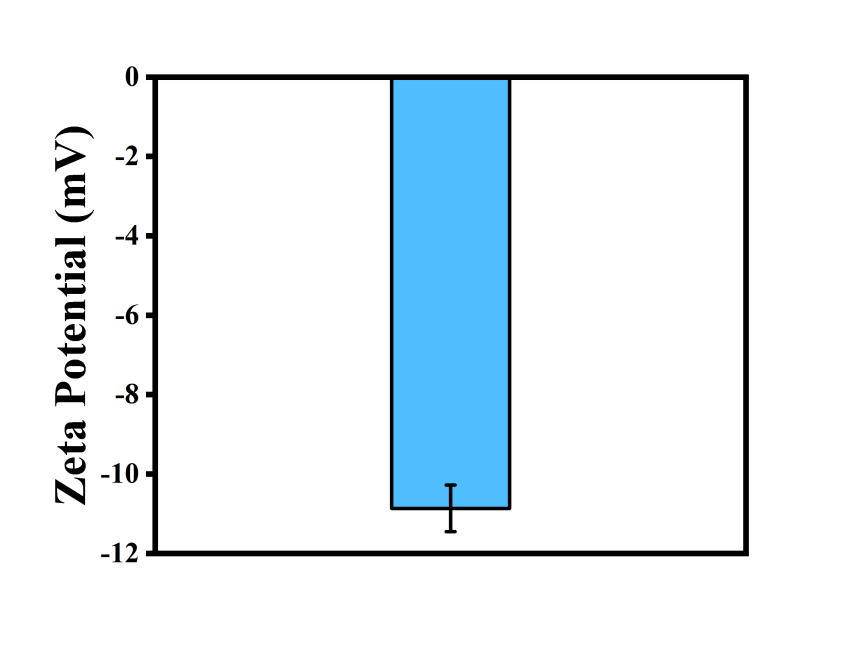


**Figure S2.** Zeta potential of CFO NPs in PBS.


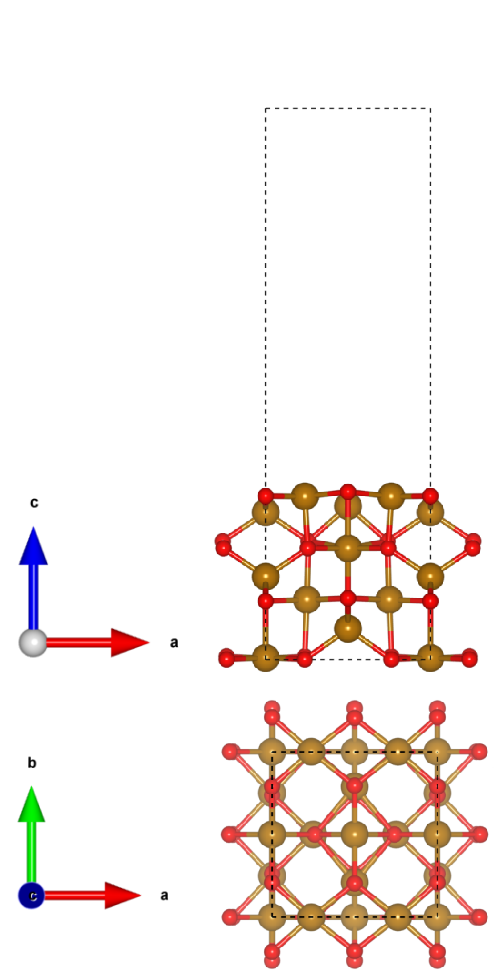


**Figure S3.** Ball-and-stick model conformation diagrams of Fe_3_O_4_(100).


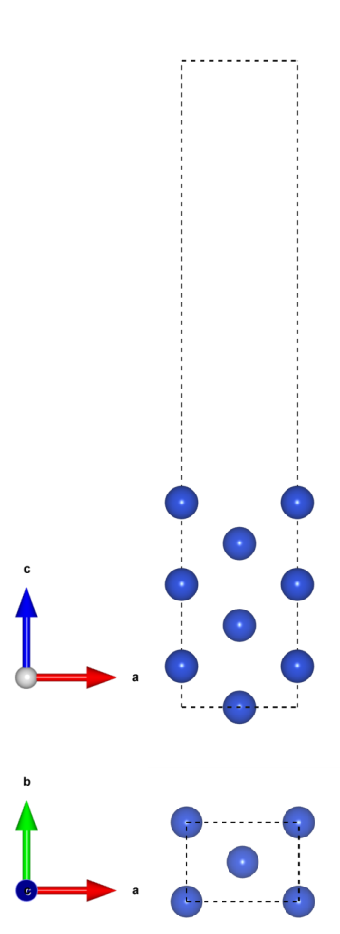


**Figure S4.** Ball-and-stick model conformation diagrams of Cu(110).


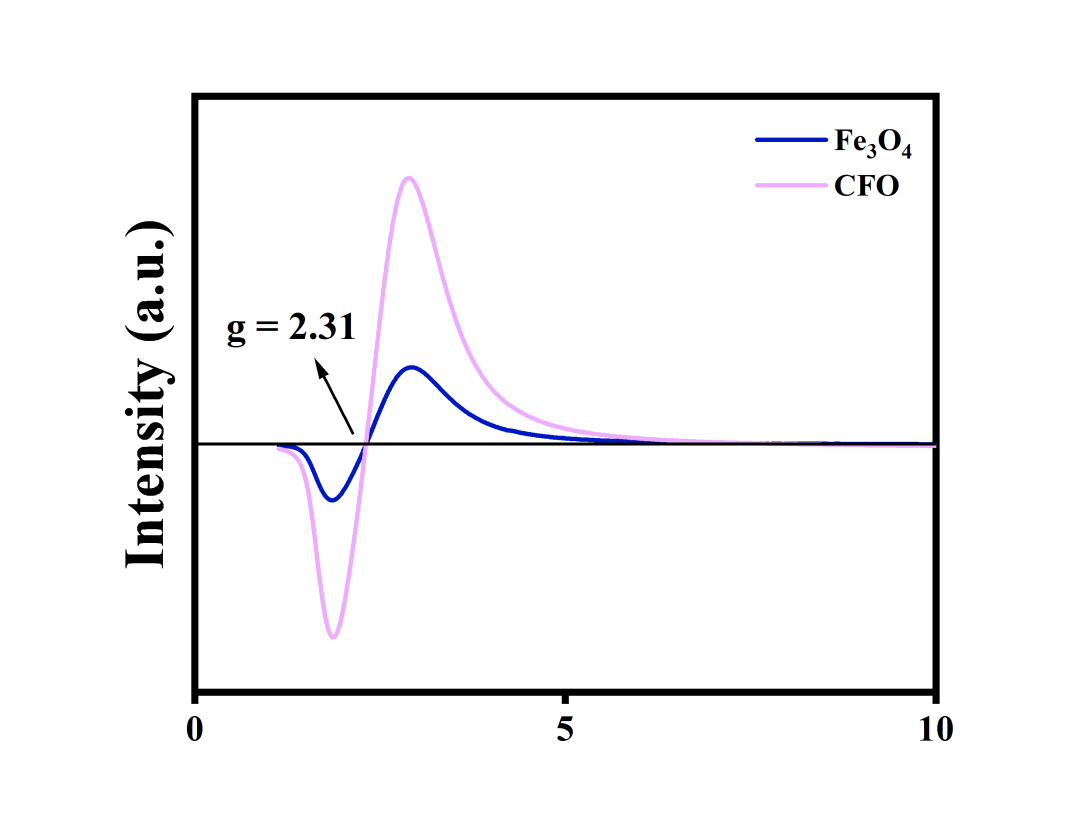


**Figure S5.** EPR spectra of ≡Fe^2+^ of CFO NPs and Fe_3_O_4_ NPs.


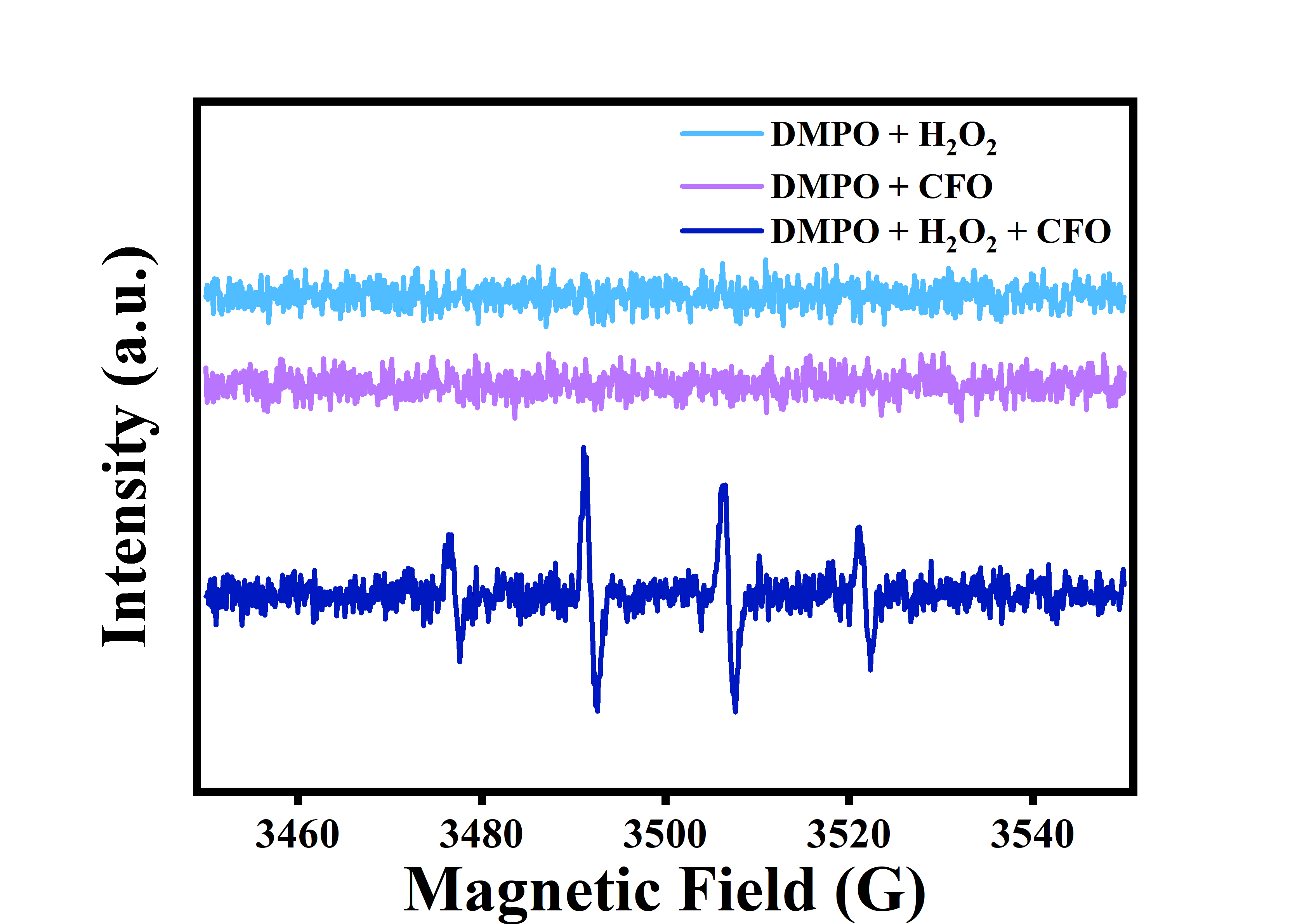


**Figure S6.** EPR spectra of CFO NPs with H_2_O_2_.


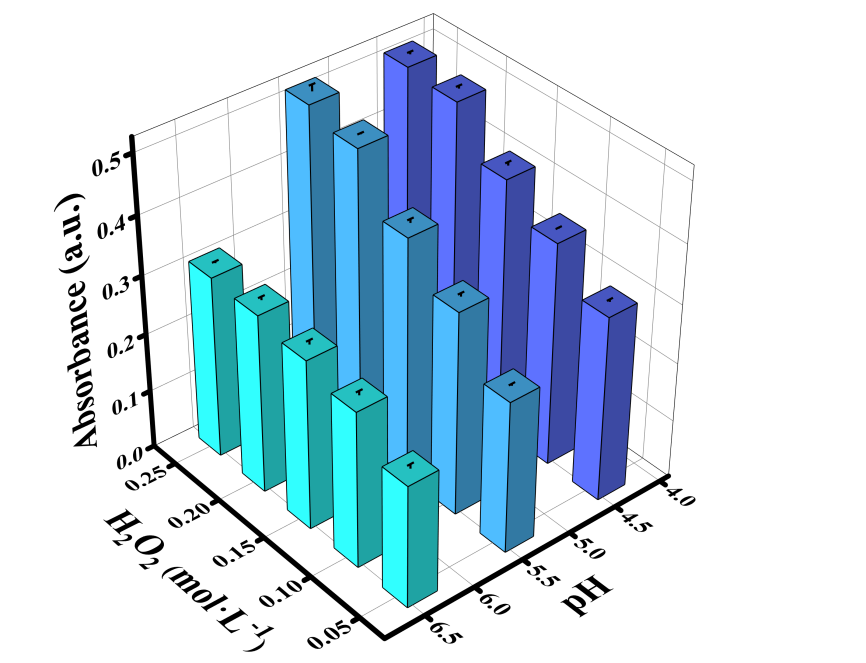


**Figure S7.** •OH generation of CFO NPs with different pH and concentration of H_2_O_2_, n = 3.


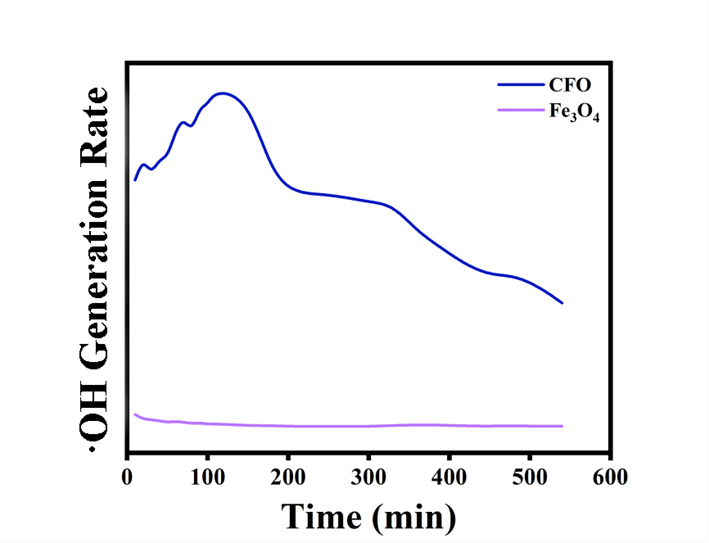


**Figure S8.** •OH generation rate of CFO NPs and Fe_3_O_4_ NPs.


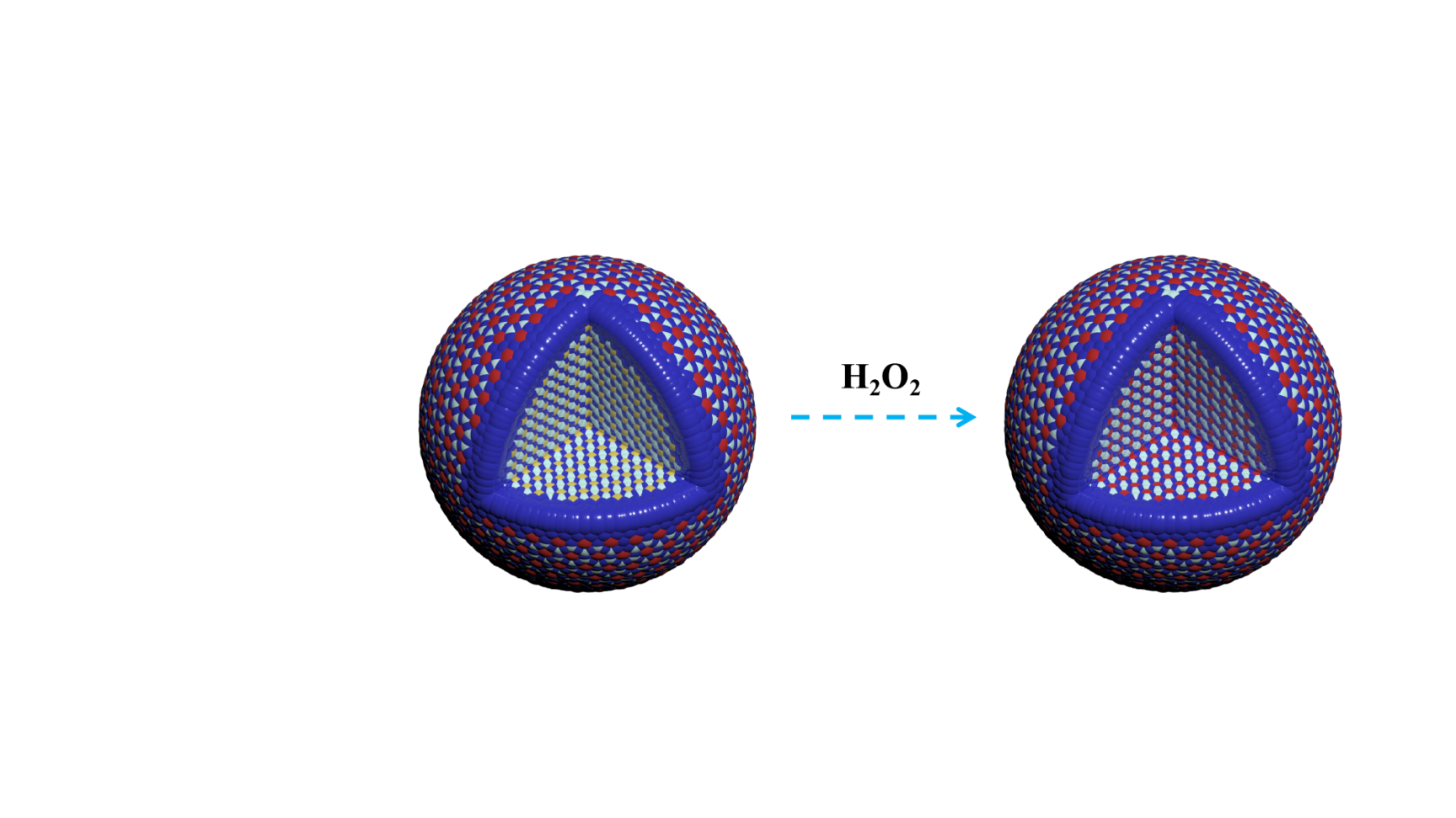


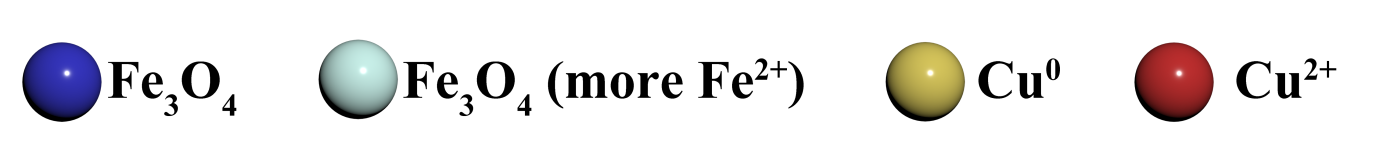


**Figure S9.** The schematic structure change of CFO NPs after incubation with H_2_O_2_.


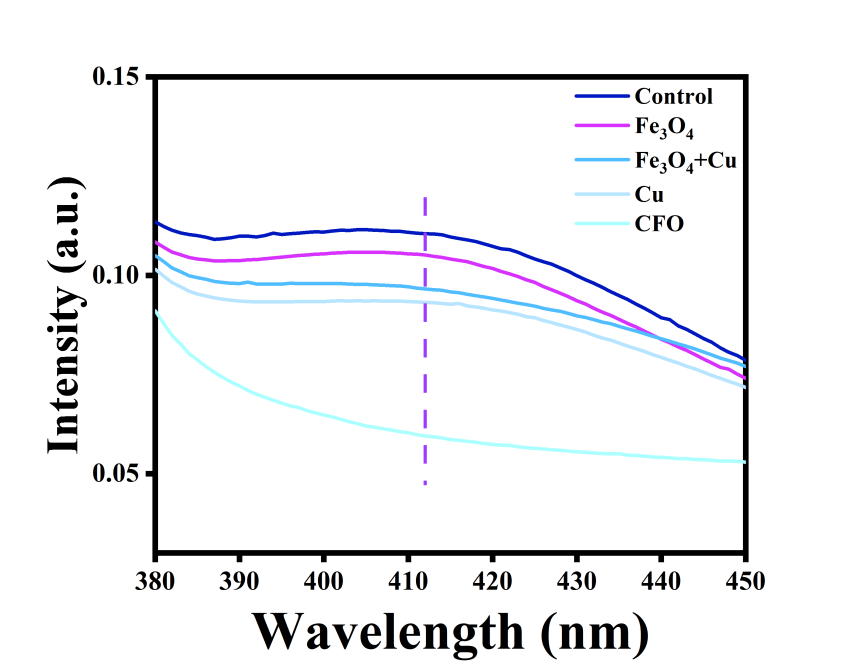


**Figure S10.** The UV-Vis spectrum of GSH consumption of different NPs. Origin GSH concentration was 1 mM. DTNB was used as the GSH indicator.


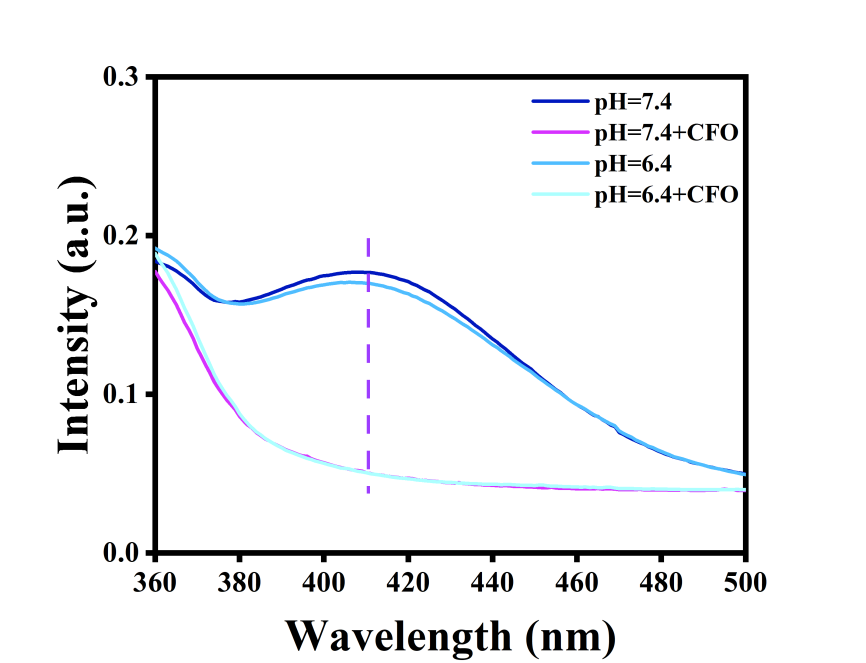


**Figure S11.** The spectrum of GSH consumption in different pH.


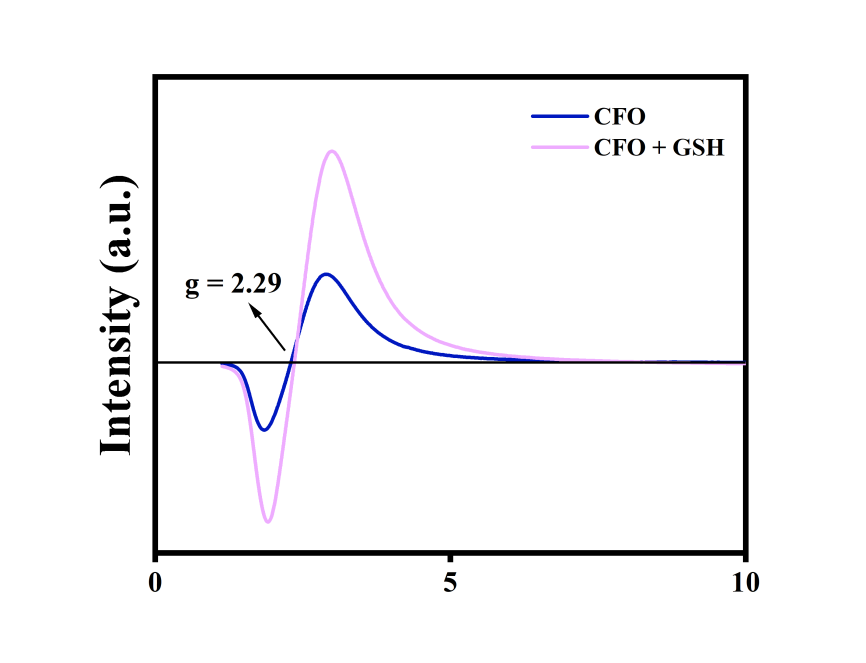


**Figure S12.** EPR spectra of ≡Cu^+^.


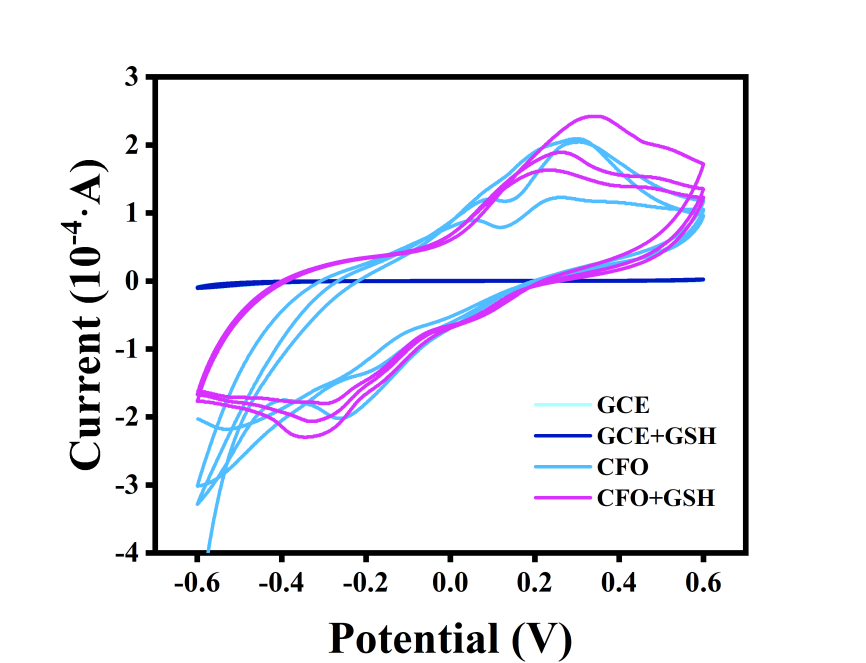


**Figure S13.** The CV curves of CFO NPs.


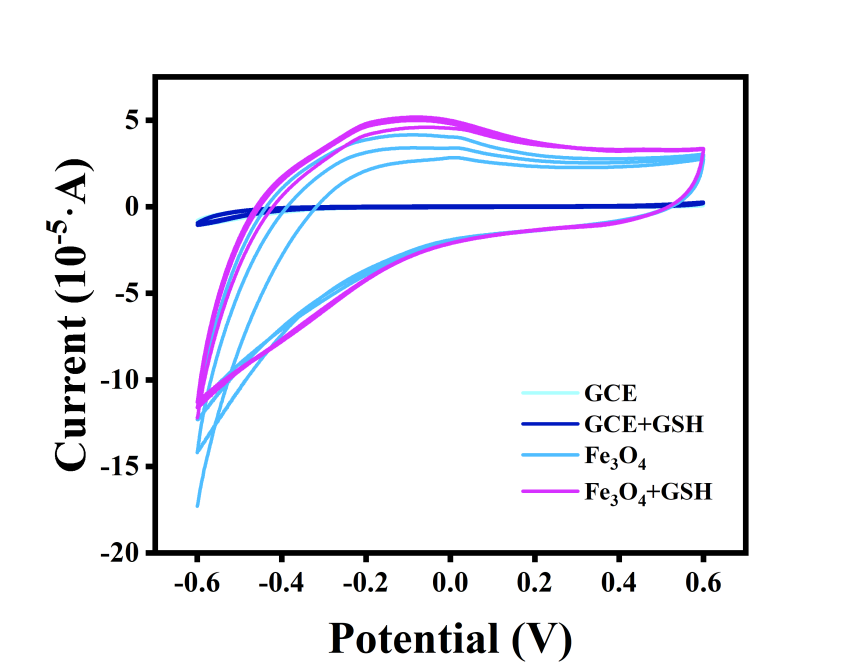


**Figure S14.** The CV curves of Fe_3_O_4._

_
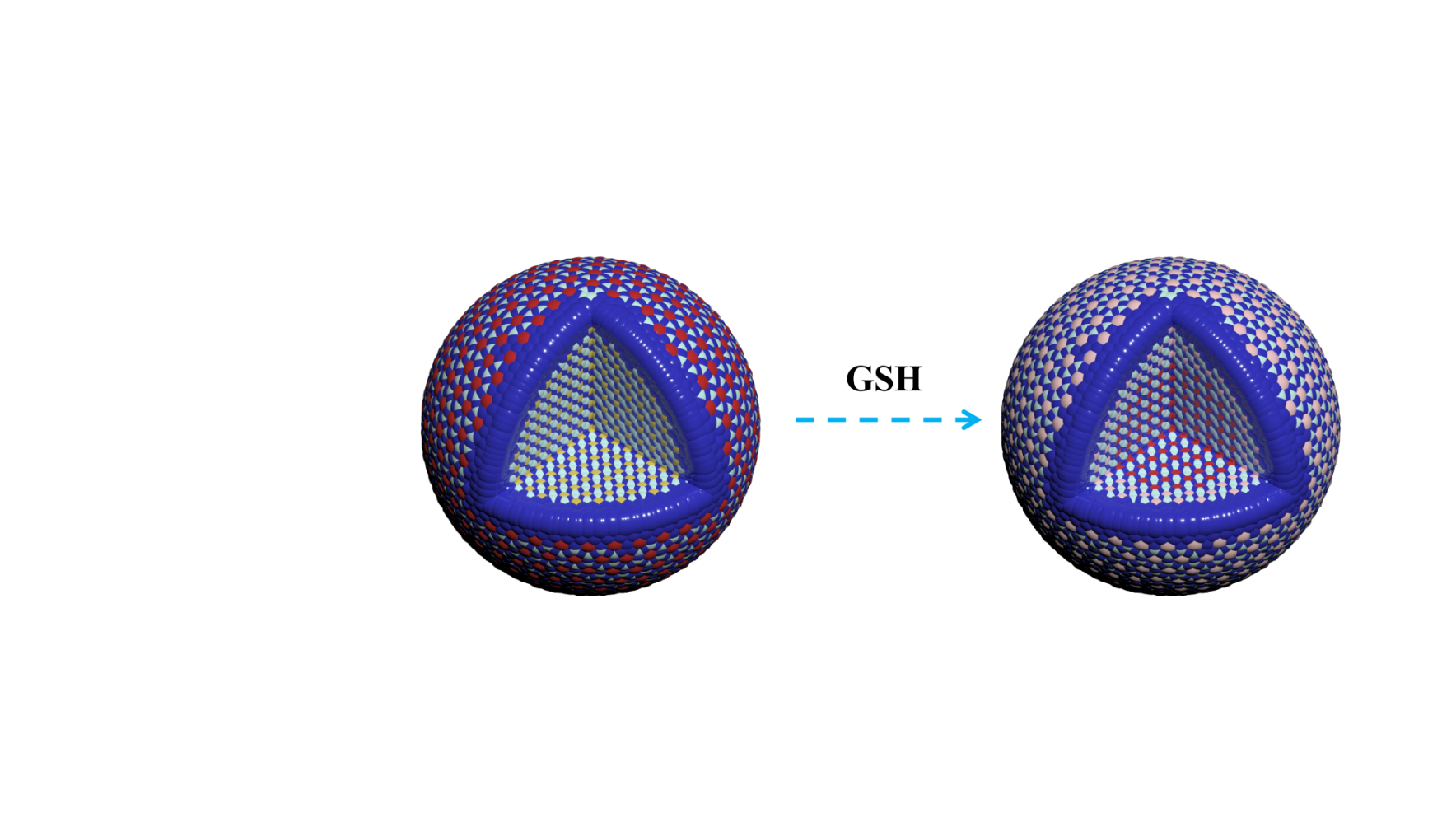
_

_
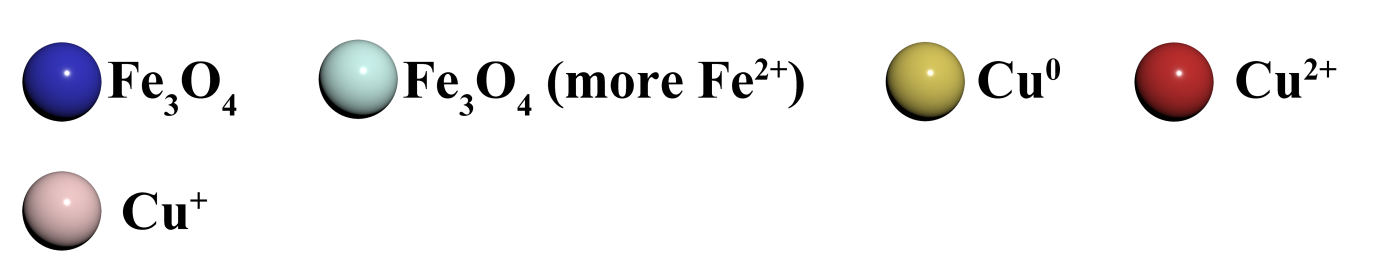
_

**Figure S15.** The schematic structure change of CFO NPs after incubation with GSH.


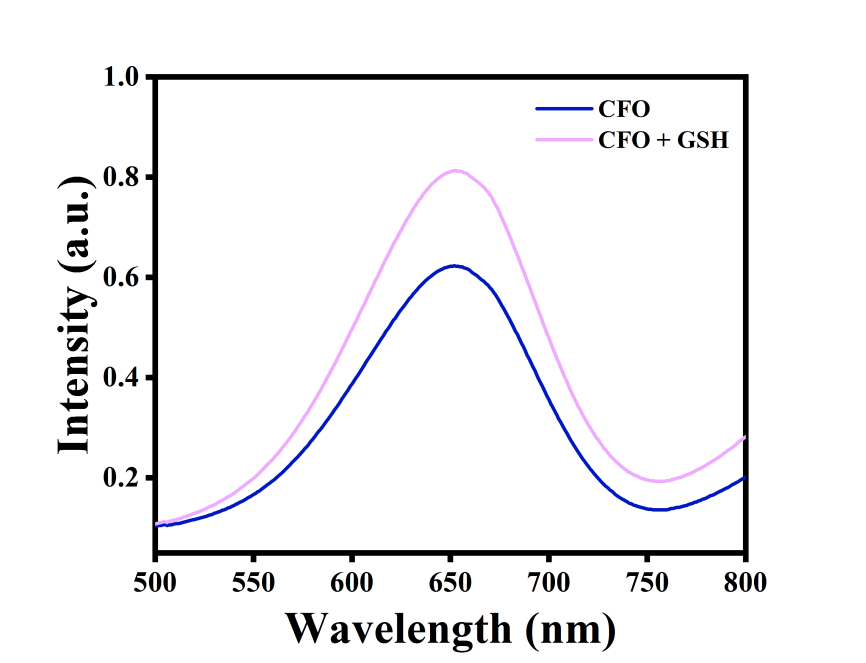


**Figure S16.** •OH generation of CFO with or without GSH treatment for 24 h. TMB was applied as the indicator. The UV-Vis spectrum was tested 2 min after the mixing with H_2_O_2_.

_
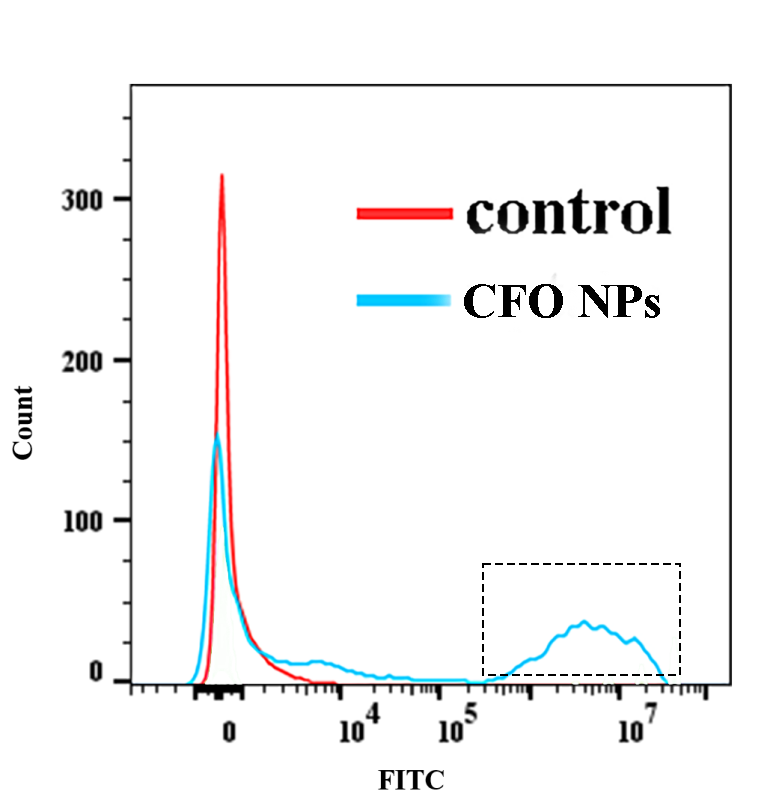
_

**Figure S17.** FITC fluorescence signal intensity of 4T1 cell after incubation with FITC labeled CFO NPs for 6 h.

_
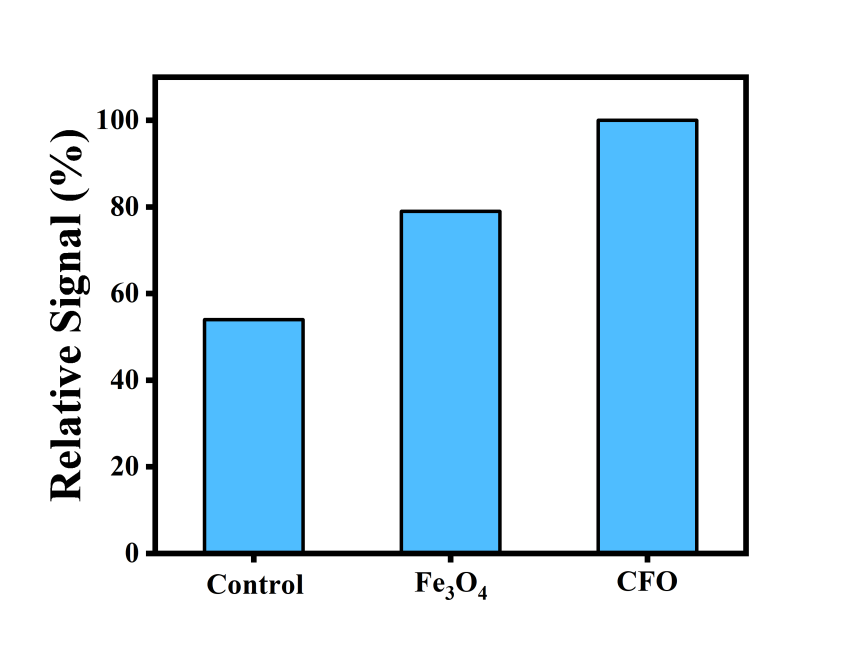
_

**Figure S18.** Quantitative analysis results of cleaved caspase3 protein.


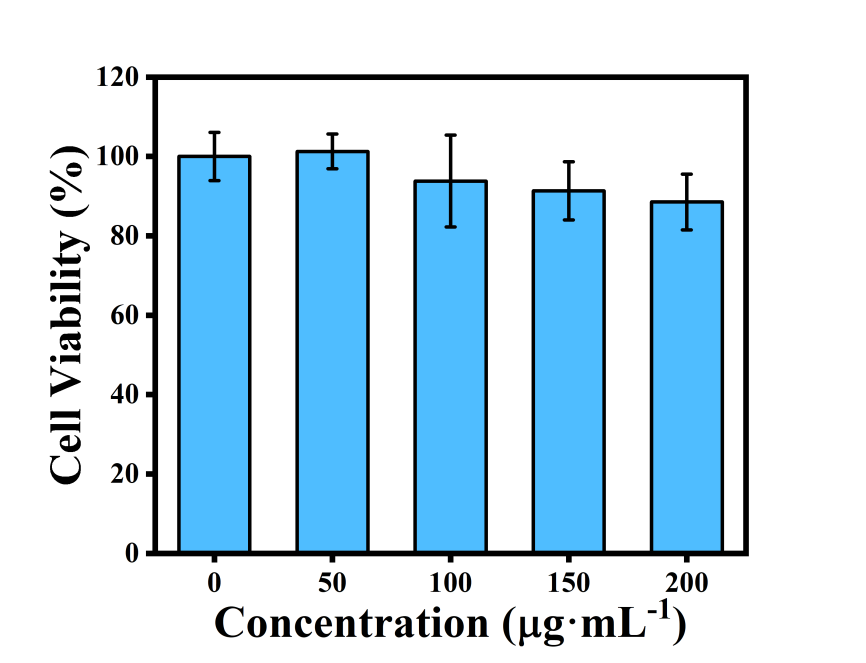


**Figure S19.** Viability of L929 cells incubated with different concentrations of CFO NPs,

n = 6, ***p < 0.001.


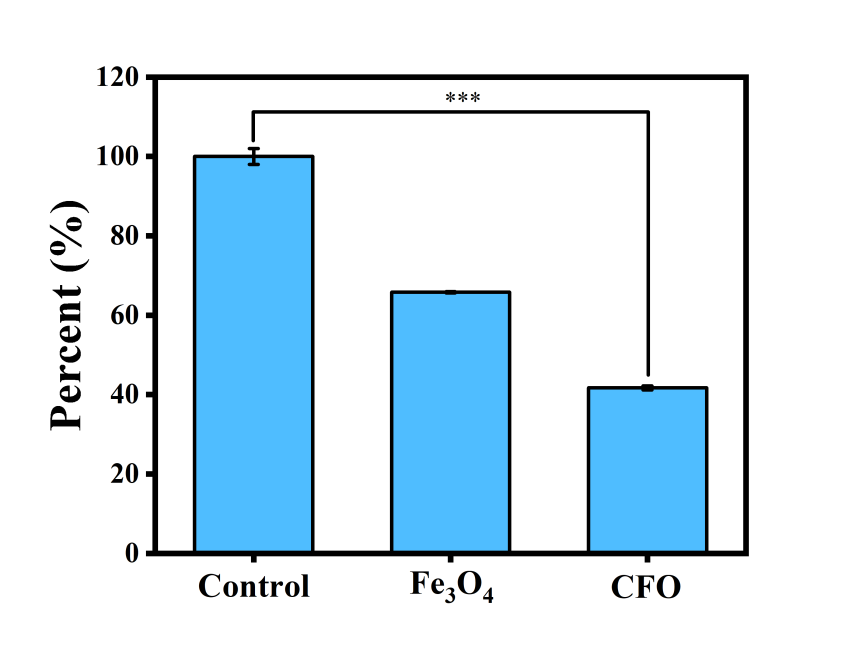


**Figure S20.** Intracellular lipid content by incubation 4T1 cells with CFO NPs or Fe3O4 NPs. n = 3, ***p < 0.001.

_
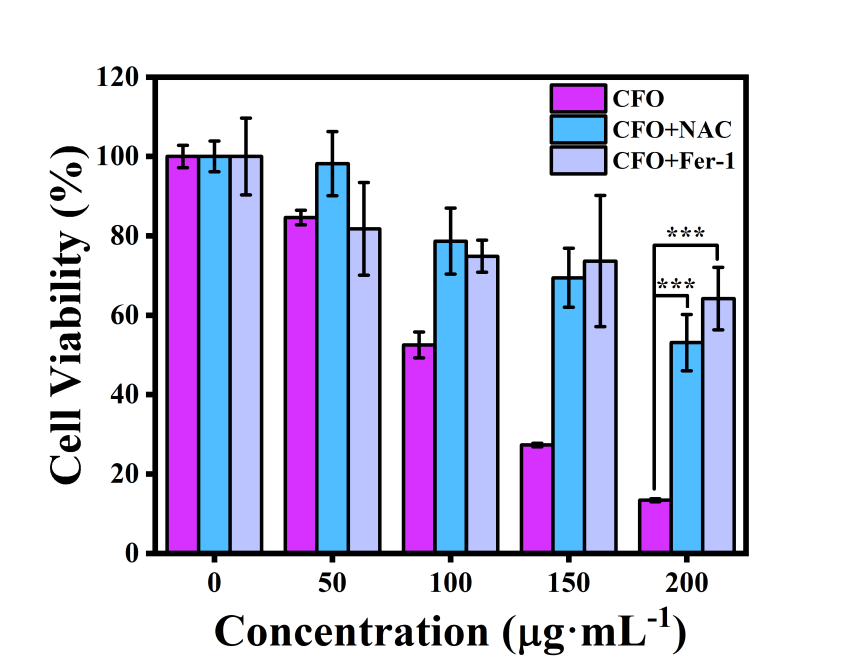
_

**Figure S21.** Viability of 4T1 cells incubated with CFO , CFO with NAC and CFO with Fer-1, n = 6, ***p < 0.001.


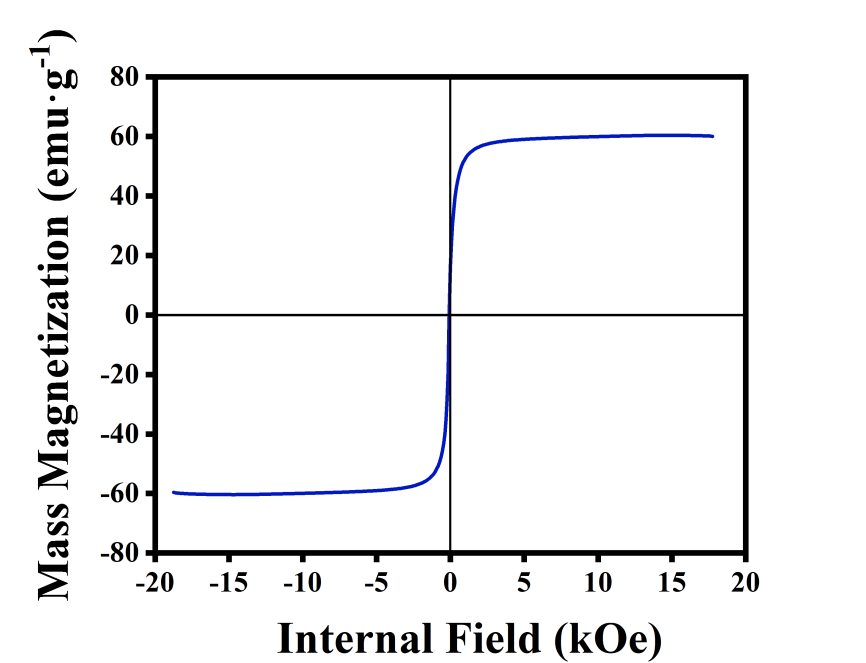


**Figure S22.** Magnetization curve of CFO NPs.


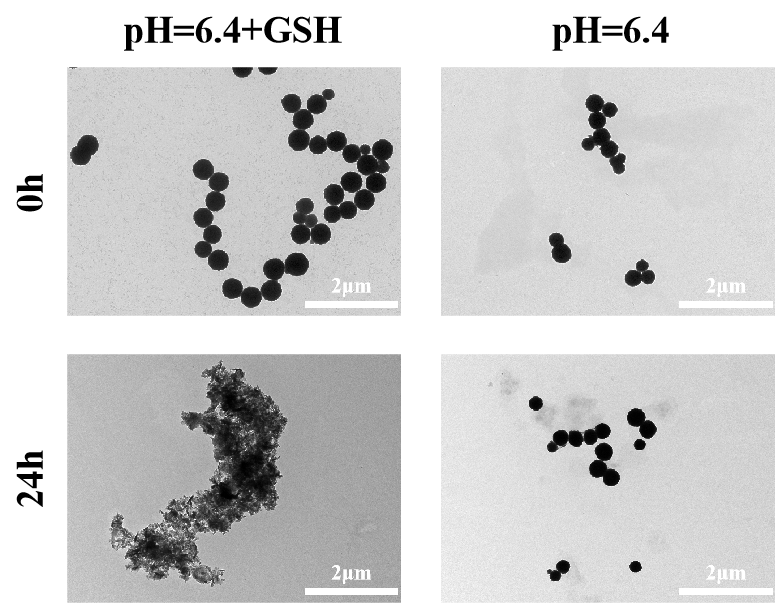


**Figure S23.** TEM images of CFO NPs with or without GSH incubating.


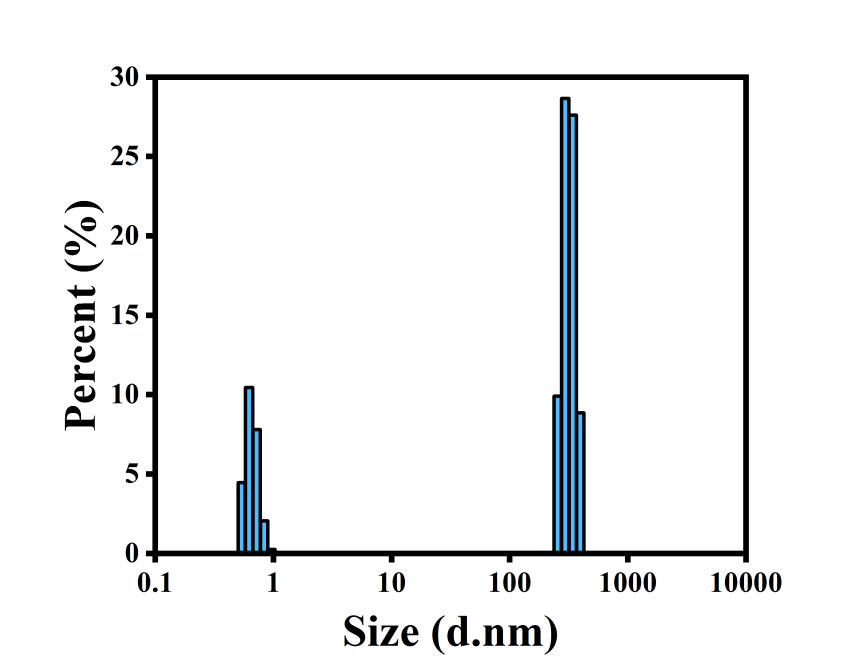


**Figure S24.** Size of CFO NPs after incubating with GSH (pH = 6.4) for 24 h.


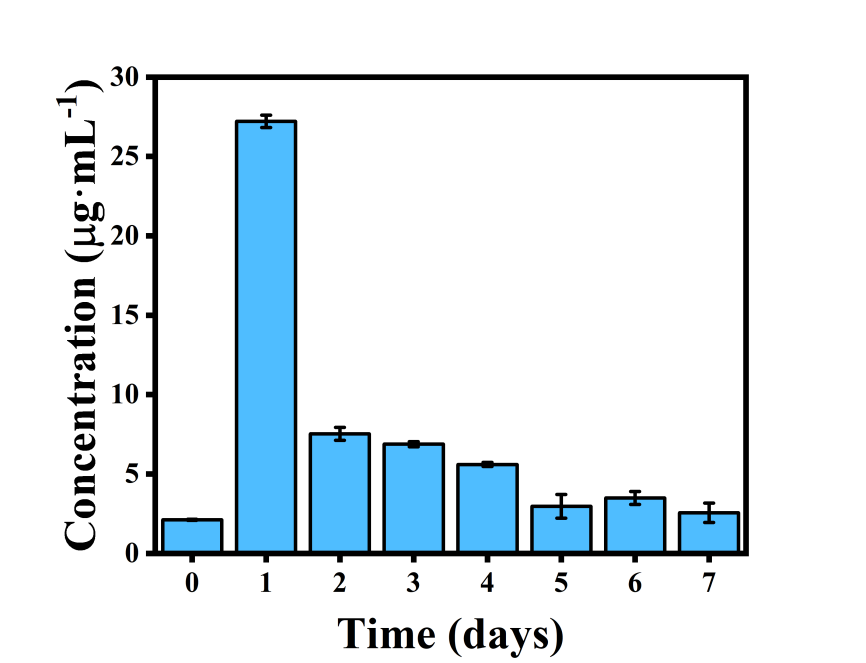


**Figure S25.** Cu content in urine of mice after injection of CFO NPs.


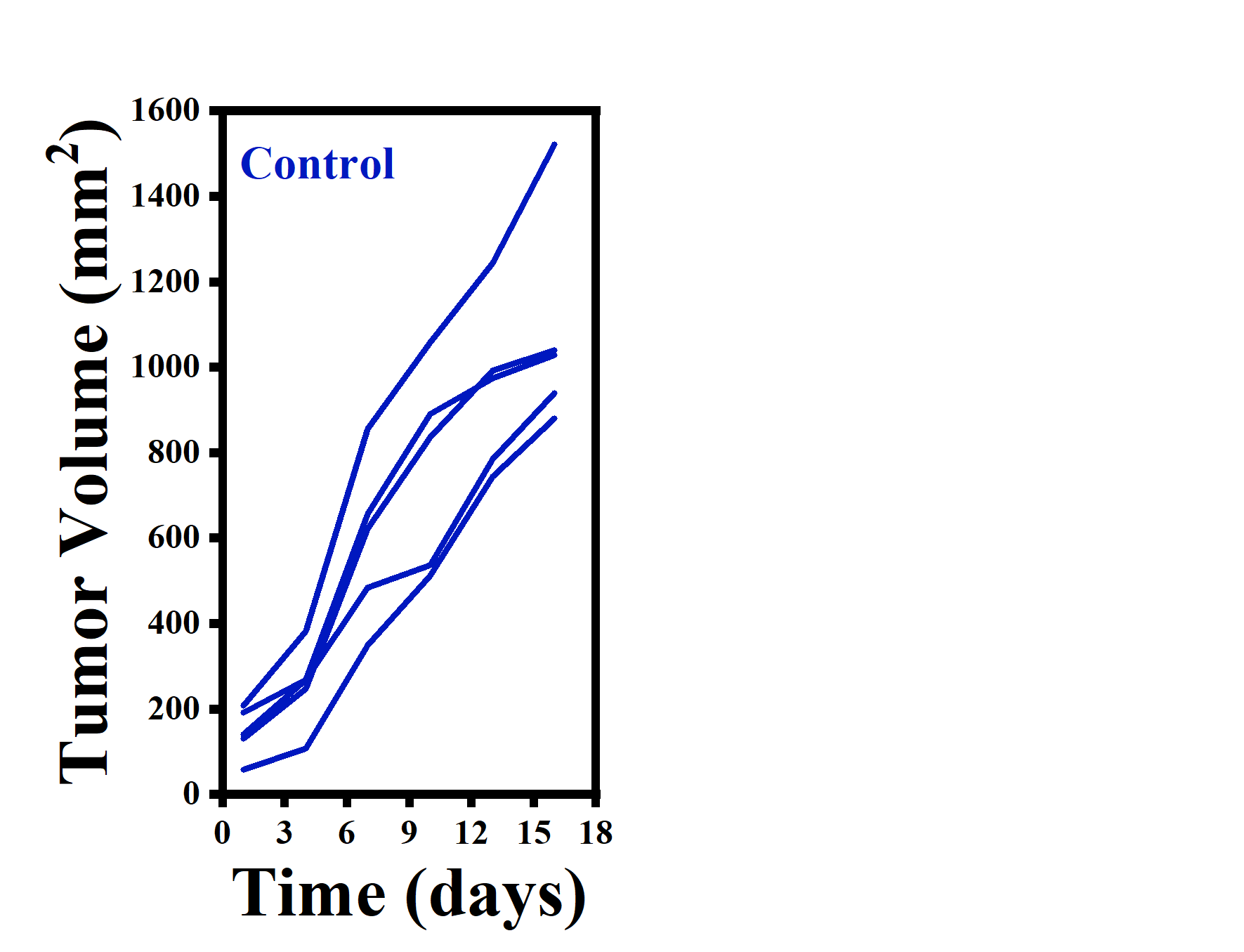


**Figure S26.** The change of tumor volume of control group.


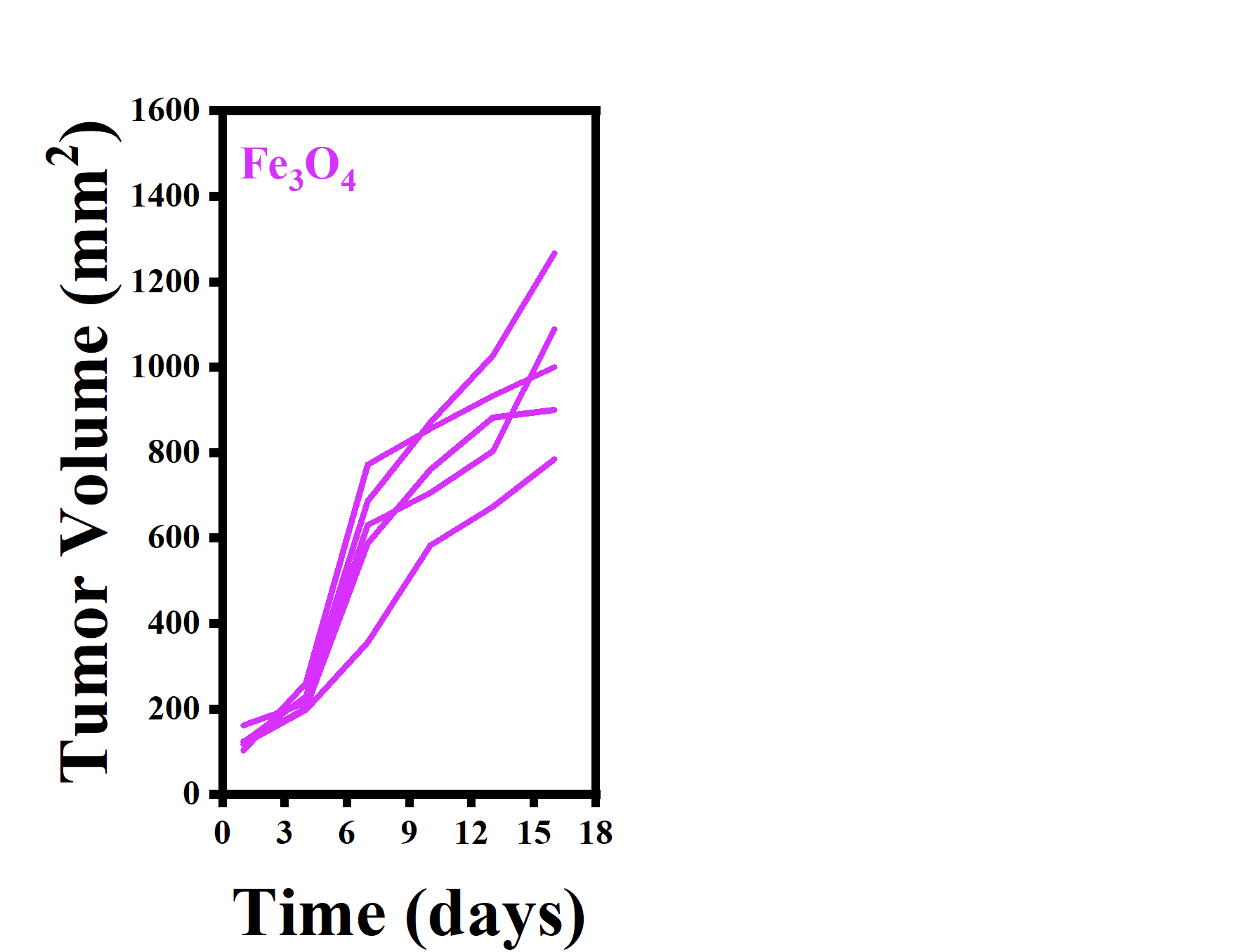


**Figure S27.** The change of tumor volume of Fe_3_O_4_ group.


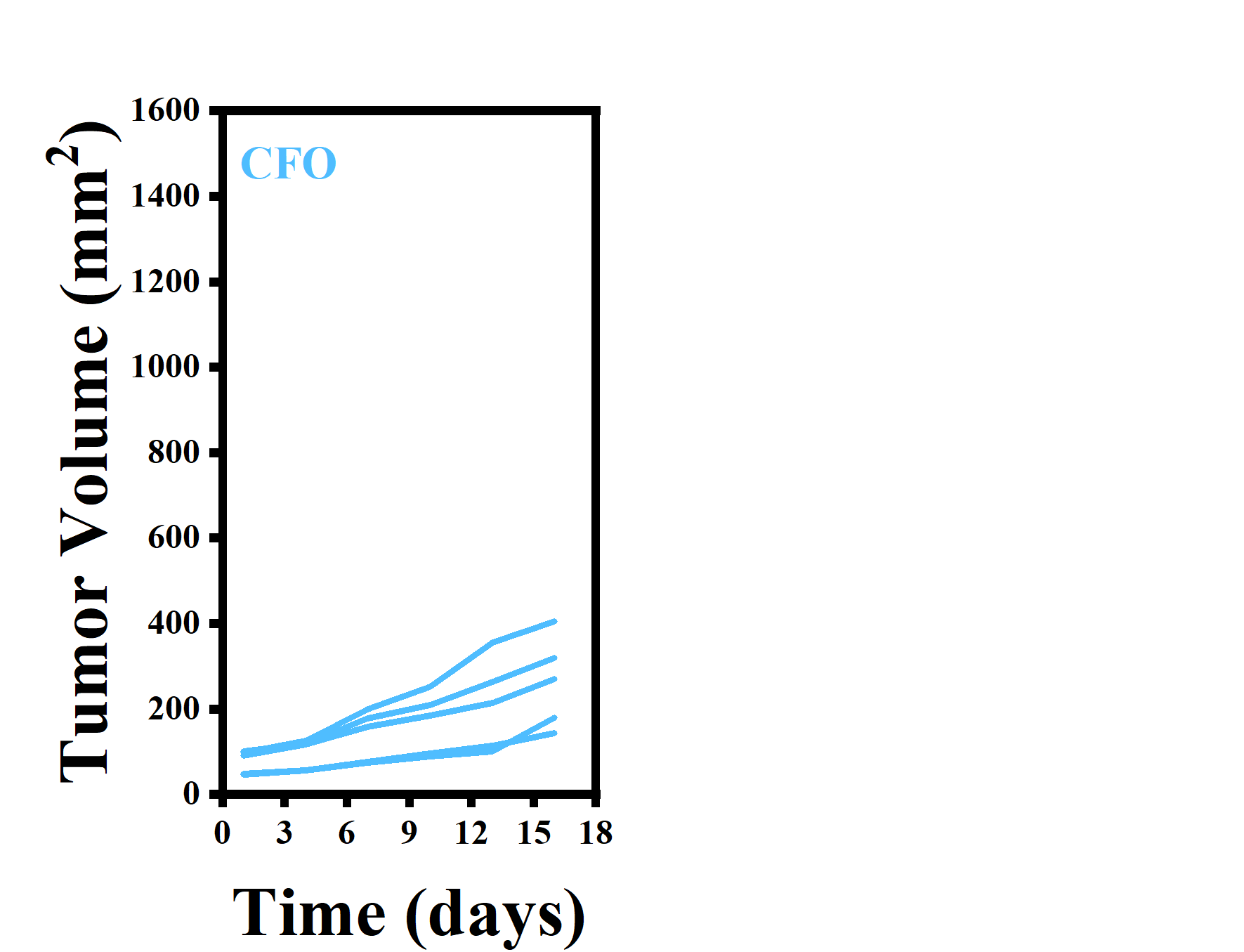


**Figure S28.** The change of tumor volume of CFO group.


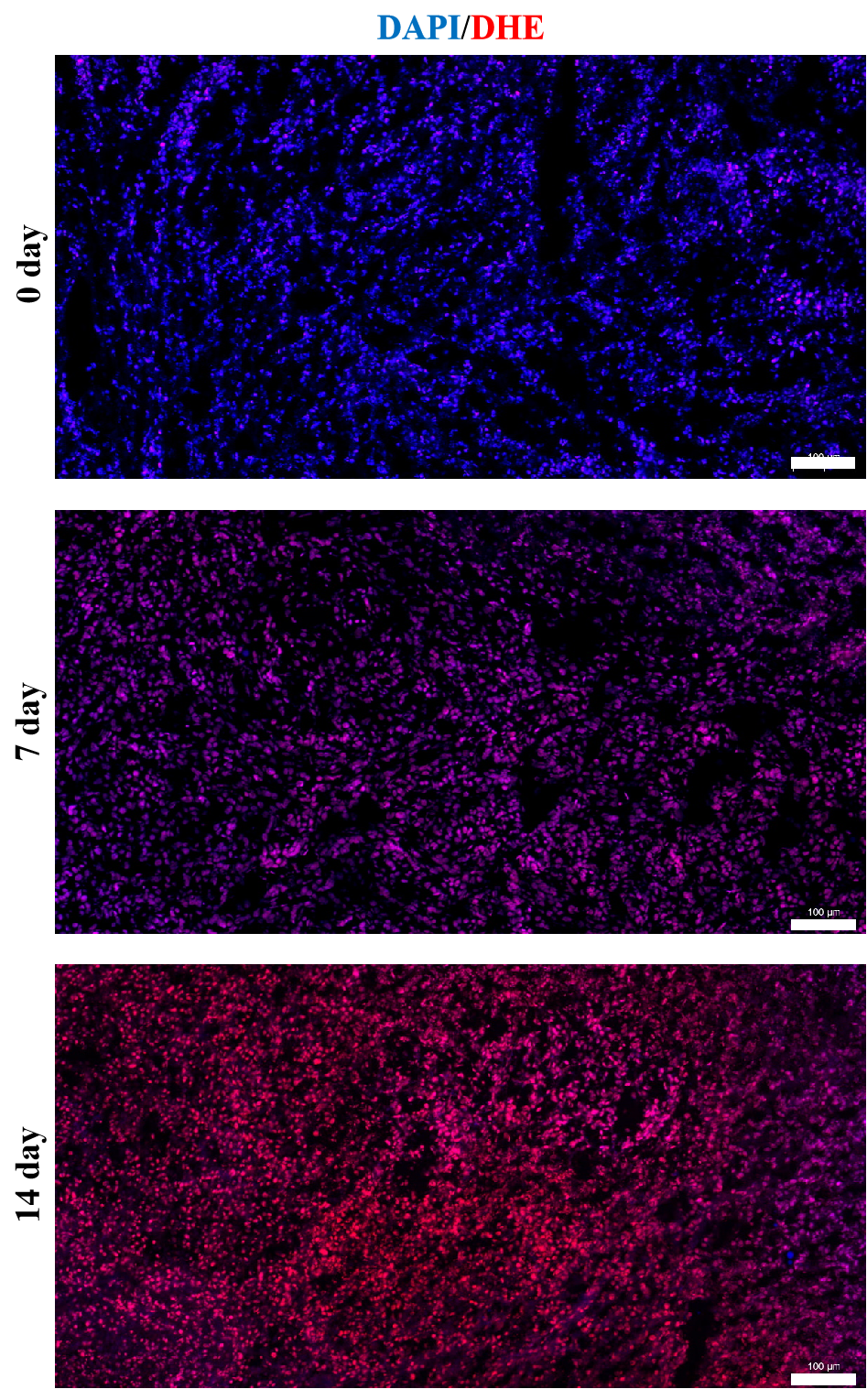


**Figure S29.** Images of DHE staining of tumor post injection of CFO NPs for different times. scale: 100 μm.
